# Rapid conversions and avoided deforestation: examining four decades of industrial plantation expansion in Borneo

**DOI:** 10.1038/srep32017

**Published:** 2016-09-08

**Authors:** David L. A. Gaveau, Douglas Sheil, Husnayaen, Mohammad A. Salim, Sanjiwana Arjasakusuma, Marc Ancrenaz, Pablo Pacheco, Erik Meijaard

**Affiliations:** 1 Center for International Forestry Research, P.O. Box 0113 BOCBD, Bogor 16000, Indonesia; 2 Department of Ecology and Natural Resource Management (INA), Norwegian University of Life Science (NMBU), Box 5003, 1432 Ås, Norway; 3 Borneo Futures project, People and Nature Consulting International, Ciputat, Jakarta, 15412, Indonesia; 4 HUTAN, Kinabatangan Orang-utan Conservation Programme, Kota Kinabalu, Sabah, Malaysia; 5 School of Biological Sciences, University of Queensland, Brisbane, QLD 4072, Australia

## Abstract

New plantations can either cause deforestation by replacing natural forests or avoid this by using previously cleared areas. The extent of these two situations is contested in tropical biodiversity hotspots where objective data are limited. Here, we explore delays between deforestation and the establishment of industrial tree plantations on Borneo using satellite imagery. Between 1973 and 2015 an estimated 18.7 Mha of Borneo’s old-growth forest were cleared (14.4 Mha and 4.2 Mha in Indonesian and Malaysian Borneo). Industrial plantations expanded by 9.1 Mha (7.8 Mha oil-palm; 1.3 Mha pulpwood). Approximately 7.0 Mha of the total plantation area in 2015 (9.2 Mha) were old-growth forest in 1973, of which 4.5–4.8 Mha (24–26% of Borneo-wide deforestation) were planted within five years of forest clearance (3.7–3.9 Mha oil-palm; 0.8–0.9 Mha pulpwood). This rapid within-five-year conversion has been greater in Malaysia than in Indonesia (57–60% versus 15–16%). In Indonesia, a higher proportion of oil-palm plantations was developed on already cleared degraded lands (a legacy of recurrent forest fires). However, rapid conversion of Indonesian forests to industrial plantations has increased steeply since 2005. We conclude that plantation industries have been the principle driver of deforestation in Malaysian Borneo over the last four decades. In contrast, their role in deforestation in Indonesian Borneo was less marked, but has been growing recently. We note caveats in interpreting these results and highlight the need for greater accountability in plantation development.

The expansion of plantations at the expense of tropical forest worries environmentalists and other observers. In particular, the rapid expansion of African oil-palm (*Elaeis guineensis*) plantations has generated alarm about the future of species- and carbon-rich old-growth and selectively logged natural forests^1^,^2^. Such concerns are particularly focused on Southeast Asia where Indonesia and Malaysia already contributed 53% and 34% (33.5 Mt and 21.2 Mt) respectively to global palm-oil production in 2013^3^. Notably, Indonesia lost 6 Mha of old-growth and selectively logged natural forests between 2000 and 2012, and surpassed Brazil in the rate of its forest clearance in 2012^4^. According to official estimates, Borneo — Earth’s third largest island with a 73.7 million hectares (Mha) landmass — supported at least 5.4 Mha of planted oil-palm in 2015^5^,^6^. Our own estimates (see results) indicate a larger planted area, i.e., 7.9 Mha of industrial oil-palm plantations for the whole of Borneo, and more in small-holder plantations: together about half of the estimated global planted area of 18 Mha^3^. Pulpwood plantations –mainly fast growing *Acacia* and *Eucalyptus* – also make a major contribution to the region’s economy. Official 2014 figures indicate that total production in the pulp and paper sector yielded an export value in excess of 5.11 billion USD and 0.61 Billion USD for Indonesia and Malaysia respectively (calculated by the authors from http://faostat3.fao.org data downloaded 2/5/2016).

In 1973, an estimated 24% (16.3 Mha) of Borneo’s land area had lost its old-growth forest cover, while old-growth forests covered 55.8 Mha (76%)^7^. Over the 1973–2015 period — the era of industrial-scale selective logging and plantation industries on the island — large tracts of species- and carbon-rich old-growth lowland forests (<500 m asl) were impacted by selective timber cutting^7^,^8^, and further degraded by illegal logging, droughts and fires^9^,^10^,^11^. Though locally present for centuries^12^, forest fires have become a large-scale cause of forest degradation and loss since the El Niño droughts of 1983^9^,^13^,^14^. Once the forest has burned, the increased risk of subsequent fires leads many forests to cycles of repeated burns^15^. Such cycles have converted millions of hectares of old-growth and selectively logged forest to degraded scrublands^15^,^16^. Some of the remaining forests were converted to industrial plantations (mainly oil-palm and pulpwood)^17^,^18^, small-scale agriculture and mining operations^19^, while some have been flooded by dams^20^,^21^.

The extent to which industrial plantations are responsible for the loss of old-growth and selectively logged natural forests in Borneo is contested. Despite many different drivers of deforestation, many non-governmental organizations (NGOs), identify industrial oil-palm plantations as the main driver of deforestation on Borneo^1^, with smallholder plantations accounting for perhaps another 35–40% of planted oil-palm^2^, and adding to deforestation pressure. The debate is not one-sided. Many commentators argue that the concerns raised over the impacts of industrial oil-palm plantations on forests are often overstated and misleading. Various industry and government representatives dispute that oil-palm plantations cause deforestation and highlight that plantations are a reasonable use of already deforested and degraded abandoned land. For example, Indonesia’s Minister for Agriculture, has stated that plantation development takes place only on abandoned land^22^. In Malaysia, the Land Development Minister of Sarawak and Malaysian Palm Oil Council chief executive officer similarly stated that oil-palm did not cause deforestation but made use of land that was already deforested^23^,^24^. The coexistence of such disparate views reflects the dearth of credible information regarding the replacement of forests by plantations.

Much is at stake. Plantations are a major element of national development for many tropical countries where labor and land costs are low. These plantations, especially oil-palm plantations, can bring significant revenues to locations where alternative sources of income are limited^2^,^25^,^26^. Planted on unused degraded lands, oil-palm has the potential to contribute more effectively to economic development and to human welfare than any comparable crop and (at least potentially) with a relatively low, albeit poorly quantified environmental cost^2^. In the order of four million people already work in the oil-palm sector in Malaysia and Indonesia^27^. At least two million more are employed by the pulpwood sector^28^. The Indonesian government has stated its intentions to be a global leader in plantation forestry with plantation areas tripling between 2010 and 2030^29^.

New plantations can either be located where they replace natural forests or where they use land already lacking such forests. The environmental and societal costs associated with plantations replacing old-growth and selectively logged old-growth natural forests, are severe^2^,^30^. In contrast, planting on already deforested land so as to avoid causing forest loss is widely viewed as desirable, and such deforestation avoiding plantings are endorsed by certification bodies; and compatible with zero-deforestation pledges^31^. Distinguishing harmful from benign planting practices has generally faltered over conflicting claims and limited evidence. Some commentators have called for a boycott of forest-harming products, while others have demanded that consumers should be permitted to choose. Such pressures have led to an EU Regulation (1169/2011), unpopular with the oil-palm industry and producer countries, requiring products containing palm-oil to be labelled so that consumers can exercise individual choice^32^,^33^. Similar issues surround the sourcing and certification of wood, paper and other tree derived products^34^,^35^, and, more recently, zero-deforestation corporate pledges.

One analysis, using FAO data, estimated that at least 56% (1.7 Mha) of Indonesia’s large-scale industrial plantations established between 1990 and 2005 replaced forests^36^. However, the reliability of these FAO datasets has been questioned^1^,^37^. Another study, based on an assessment of deforestation within government-registered concession boundaries, estimated that 1.6–1.9 Mha of forest were cleared for industrial oil-palm and pulpwood plantations between 2000 and 2010 in Indonesia (1.1 Mha and 0.6 Mha in Indonesian Borneo)^17^. However, using available concession maps to link deforestation to specific companies is frequently disputed by those implicated due to inaccurate maps, overlapping claims and the activities of others; nor can it account for clearing outside concession boundaries^38^.

Two studies in Indonesian Borneo (Kalimantan) have reconstructed the expansion of industrial oil-palm plantations between 1990 and 2010 using medium-resolution LANDSAT satellite imagery^39^,^40^ regardless of whether the planted palms were inside or outside concessions. These reconstructions are possible due to the ease with which industrial plantations can be identified on imagery, (see also methods; small-holder oil-palm in contrast cannot be consistently distinguished using LANDSAT). Both studies found that nearly a quarter of plantations were outside government-registered concessions, validating the need to look beyond concessions to measure impacts of this industry on forest cover. The first study defined *Forest* as old-growth and selectively logged natural forests and estimated that 43% (1.2 Mha) of industrial oil-palm plantations were developed at the expense of forests^39^. The second study included man-made agro-forests in the definition of *Forest* and estimated that 90% of industrial oil-palm plantations were developed at the expense of forests^40^. However, neither study distinguished forests cleared directly for oil-palm from forests initially cleared for other reasons that were then later planted with oil-palm. This oversight matters to those seeking to improve land-use practices.

To measure the area of forest initially cleared for other reasons, and later converted to industrial plantations, we would need to know if the forest was cleared independently of (i.e., before) any plan by the ultimate plantation concession holder, and any other associated beneficiaries, to develop the plantations. Unfortunately, the governments of Indonesia and Malaysia insist that licensing data remain confidential. The partial data that has entered the public sphere is not representative and generally lacks the full list of licensing dates for each concession^41^,^42^. Specifically for Indonesia, the national Land Registry does not hold full records of overlapping claims. As already noted, a comprehensive analysis of land rights and responsibilities must account for overlapping claims and activities: communities occupy concessions, while companies expand outside their boundaries^38^,^43^. Such an evaluation is not possible with available information.

We sought an approach to help distinguish harmful from benign planting practices. Our proposal is based on the time required for forest conversion. Time-series satellite imagery allows us to measure objectively and consistently, within defined limits, the delay between forest clearance and plantation development. We reasoned that, as a statistical generalisation, industrial plantations developed rapidly after forest clearance are likely to be responsible for that clearance (“harmful”), while the longer the delay between forest loss and plantation development the more likely such plantings are actively avoiding direct deforestation (“benign”). Interviews with fourteen senior oil-palm managers at a meeting of oil-palm executives and NGOs in Singapore in May 2015 indicated that forest conversion to industrial oil-palm is usually rapid, and occurs within two to three years. (Indeed, our own provisional analyses, indicated that of forests areas converted to plantation within five years of clearing, most is replanted within just one to three years, see also Figure S2c for a later summary of this pattern).

Here, we mapped the Borneo-wide decline in forest area and the concomitant expansion of areas developed (or under development) by plantation companies (hereafter called ‘industrial plantations’) in approximately five-year increments over six consecutive periods between 1973 and 2015 using time-series LANDSAT satellite imagery (See Methods). We defined *Forest* as a natural forest that has remained in sufficiently good condition to be seen as intact or nearly intact on LANDSAT satellite imagery – this includes old-growth and selectively logged forest (*Dipterocarps* and *Kerangas* on dry mineral soils, and on fresh-water and peat swamps as well as mangroves), and possibly some forest mildly impacted by ground fires. Our definition includes selectively logged-over forests but excludes secondary forests that regrow after forest clearance, agroforests and other planted forests. Old-growth and selectively logged forest represent the most biologically diverse ecosystems on Borneo.

For each industrial plantation, we determined in which previous period forest was present and then cleared. We defined *Rapid Conversion*, as those plantations developed within five years of forest clearance (our smallest interval), i.e. the areas where forest was still standing five years before development. With this rapid conversion, we postulate that most plantations developed within five years of clearance are responsible for that clearance. Conversely, we identified plantations established on land that has been free of forest for >5 yrs, >10 yrs, >15 yrs, or since 1973 (our baseline year). These plantations are less likely to be responsible for the initial deforestation. We underline that this will not be true in every case, and that this method cannot attribute blame in specific cases. A five-year interval is a pragmatic choice (and we also consider other intervals), we don’t expect a sharp threshold and accept ambiguity in individual cases. We can imagine scenarios in which permission is granted to convert forest to plantations after which it may first be logged, and perhaps burned and degraded long in advance of any planting due to various planned or unplanned delays. If these patterns differ by region or period it would influence our comparisons. We return to these caveats in the discussion.

Our approach is an advance on previous studies because it provides the first systematic Borneo-wide estimates of forest area rapidly converted to industrial plantations, including oil-palm and pulpwood, over four decades, and the first estimates of plantations established on land that lacked forest cover long before establishment. It is also the first to compare rapid conversion against the island-wide deforestation across four consecutive periods spanning 42 years, and to reveal how the share of rapid conversion to total deforestation evolved over time in Indonesia and Malaysia, the world’s largest producers of palm-oil and globally important producers of pulpwood.

## Results

### Borneo-wide deforestation

**Figure 1:**
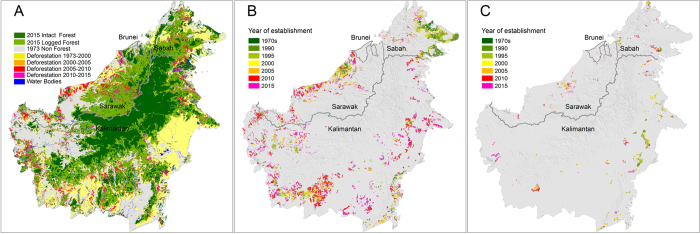
Land use and land cover change in Borneo (1973–2015). (**A**) Total deforestation (18.7 Mha) and remaining old-growth and selectively logged forest in December 2015; (**B**) The expansion of industrial oil-palm plantations (7.8 Mha); (**C**) The expansion of industrial pulpwood plantations (1.3 Mha). Maps created using *ArcMap* v10.2.2 geospatial processing program http://www.esri.com/software/arcgis/arcgis-for-desktop.

In 1973, old-growth forests covered 55.8 Mha (76%) of Borneo’s land area^7^. An estimated 18.7 Mha (34%) were cleared between 1973 and 2015, with 14.4 Mha in Indonesian Borneo (Kalimantan) and 4.2 Mha in Malaysian Borneo (Sabah and Sarawak), respectively (Fig. 1A; Table 1). Less than 46,636 ha were lost in Brunei Darussalam during this 42 year period. Seventy five percent (13.9 Mha) of this Borneo-wide deforestation occurred before year 2000, with 82% (11.5 Mha) and 53% (2.4 Mha) in Indonesian and Malaysian Borneo, respectively. We find that approximately, 83,362 ha of forest have been permanently flooded by the creation of the hydroelectric power dams of Bakun and Murum between 2011 and 2014 in Sarawak^20^,^21^.

### Expansion of industrial plantations over time

**Figure 2:**
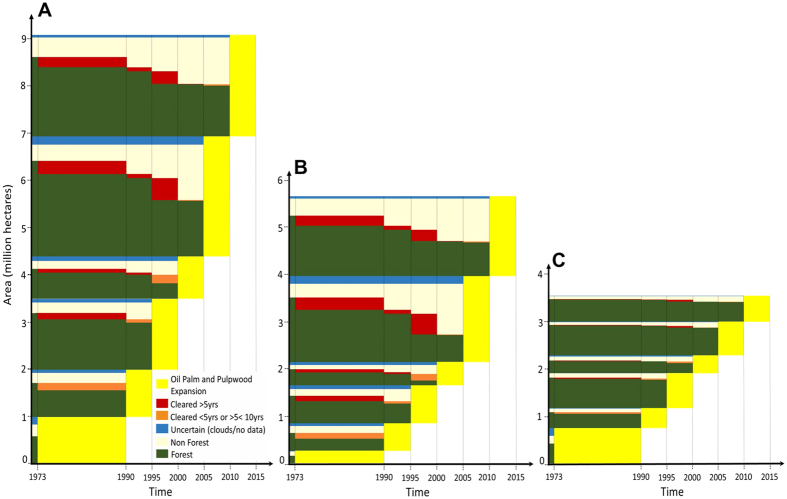
The expanding area (9.1 Mha; yellow blocks) of industrial plantations (oil-palm and pulpwood) in six time periods from 1973 to 2015 in Borneo (**A**), Indonesian Borneo (**B**), and Malaysian Borneo (**C**). The decline in forest area (dark green) since 1973, and prior to plantation establishment (yellow) is shown to the left of each yellow block in each time period. The changes reveal when the land was forest (green), non-forest (light brown) or had been cleared (red and orange) prior to plantation development (yellow). Areas cleared more than five years prior to plantation establishment are shown as red blocks. Areas where forest was cleared less than five years or more than five years, but less than ten years before plantation establishment are shown as orange blocks. The areas where the dark green blocks (forest) touch the yellow blocks are areas where forest was cleared less than five years prior to plantation establishment (rapid conversion). The blue blocks indicate areas of uncertainty, where we could not define clear land cover transitions, because of either cloud cover or lack of imagery.

Industrial plantations expanded by 9.1 Mha (7.8 Mha oil-palm; 1.3 Mha pulpwood) between 1973 and 2015 (Fig. 1B,C; Table 1; see also Table S1a,b for oil-palm and pulpwood, separately). The total area developed as industrial plantations reached 9.2 Mha in 2015 (minimum size of a plantation = 90 ha; max = 560,000 ha; mean = 4,600 ha) or 12% of Borneo’s land area. More than a half of these plantations (4.8 Mha) were planted between 2005 and 2015. Almost 70% of these recent plantings are oil-palm plantations in Indonesian Borneo (3.3 Mha since 2005; Table S1a). In contrast, plantation expansion in Malaysian Borneo has been relatively constant over time (yellow blocks in Fig. 2B,C). Industrial plantations covered only 2,625 ha in Brunei Darussalam in 2015. The estimated accuracy of our oil-palm static and dynamic maps ranged between 88–96% depending on the year (See Tables S2–S6 for full accuracy reports). Our planted oil-palm area is 4–12% larger than the area reported by two published studies in Kalimantan^39^,^40^ (Figure S1). There are no published pulpwood maps to compare our results against.

### Forest area rapidly converted to industrial plantations

**Figure 3:**
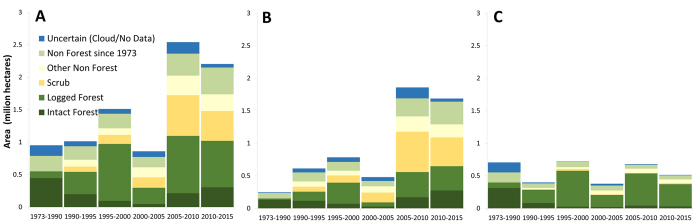
The expanding area (9.1 Mha) of industrial plantations (oil-palm and pulpwood) in six time periods from 1973 to 2015 with vegetation cover of the land just before observed conversion to plantations in Borneo (**A**), Indonesian Borneo (**B**), and Malaysian Borneo (**C**). Intact Forest: pristine old-growth forests. Logged Forest: old-growth forests that have lost their original structure and canopy cover through industrial-scale selective timber harvest at some point since 1973, indicated principally by the construction of logging roads. Scrub: old-growth forests impacted by drought and fire; these burn/drought scars tend to recover slowly. They are vulnerable to further burning and conversion to short vegetation follows; hence they appear as “deforested” in satellite assessments (see also methods). Non Forest since 1973: areas that have been cleared before 1973. Other Non-Forest: areas that have been cleared after 1973, but not converted to scrubs. We recognize that Non Forest since 1973 and Other Non-Forest may include secondary forests: young-growth, forest fallow or agro-forest.

Approximately 7.0 Mha (76%) of the total area of industrial plantations in 2015 (9.2 Mha), were old-growth forest in 1973, of which 4.5 Mha had been planted rapidly, i.e. within five years of forest clearance (see the green blocks touching the yellow blocks in Fig. 2). See also Figures S2a,b for oil-palm and pulpwood, separately. Forest area loss peaked one to three years prior to planting in forest areas rapidly converted to plantations (Figure S2c). If we consider the 0.3 Mha of forest area (orange blocks in Fig. 2) where there is some chance that forests may have been converted within five years, or between five and ten years as uncertain, we can infer that the oil-palm and pulpwood industries appear responsible for the clearance of 4.5–4.8 Mha of forest (3.7–3.9 Mha oil-palm; 0.8–0.9 Mha pulpwood) across Borneo over the period 1973–2015. This implies that rapid conversion to industrial plantations represents 24–26% (20–21% oil-palm; 4.3–4.8% pulpwood) of all post-1973 deforestation in Borneo (18.7 Mha). For Indonesian and Malaysian Borneo, these figures differ markedly: 15–16% and 57–60%, respectively. We note that at least two-thirds of the forest area lost to plantations had been selectively harvested for timber (logged) prior to clearance in all regions (72%, 63% and 78% for Borneo, Indonesian and Malaysian Borneo, respectively; Fig. 3). See also Figures S3a,b for oil-palm and pulpwood, separately.

**Figure 4:**
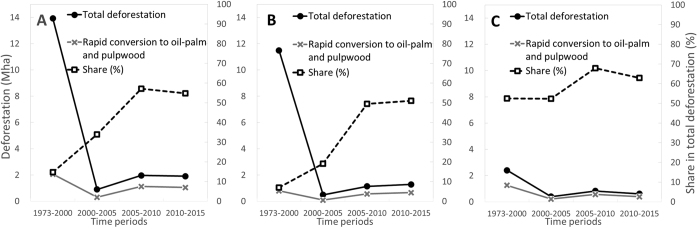
Role of industrial plantations (oil-palm and pulpwood) in deforestation by period for Borneo (**A**), Indonesian Borneo (**B**) and Malaysian Borneo (**C**). On the primary axis (left Y axis), the grey solid line indicates the area of forest rapidly converted to industrial plantations (i.e. within five years of clearance), while the black solid line indicates the total area of deforestation by time period on Borneo (see also Fig. 1A). On the secondary axis (right Y axis), the dashed line represents the share of rapid conversion in total deforestation, expressed in percentage terms.

Looking at each observation period, the combined share of old-growth and selectively logged forest rapidly converted to industrial plantations (i.e. within five years of clearance) to total deforestation in Borneo has increased over time: from 15% in 1973–2000, to 34% in 2000–2005, to 55–57% since 2005 (Fig. 4A). In absolute terms, 2.2 Mha of forest have been rapidly converted to industrial plantations from 2005 to 2015 across Borneo. This figure is 1.2 Mha for Indonesian Borneo. Rapid, within-five-year, conversion in Indonesian Borneo increased from 7% in 1973–2000 to 51% in 2010–2015 (Fig. 4B). In Malaysian Borneo, this share has always surpassed 52%, peaking at 68% in 2005–2010 (Fig. 4C). See also Figures S4a,b for oil-palm and pulpwood, separately.

### Plantations developed on lands that lacked forest cover long before establishment

**Figure 5:**
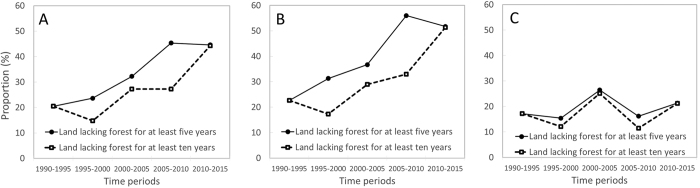
Proportion (in area terms) of industrial plantation (oil palm and pulpwood) established on land that lacked forest cover for at least five years (solid line) or at least ten years (dashed line) prior to planting, for Borneo (**A**), Indonesian Borneo (**B**) and Malaysian Borneo (**C**).

Approximately 1.8 Mha (20%) of the total area under industrial plantations in 2015 (9.1 Mha) used lands cleared before 1973 — an estimated 24% (16.3 Mha) of Borneo already lacked forest cover in 1973 (Table 1; Fig. 1). Of the 7.0 Mha old-growth forest area replaced by plantations between 1973 and 2015, approximately 1.8 Mha (the red blocks in Fig. 2; Table 1) had been cleared more than five years before plantations were visible. Therefore, 3.7 Mha (41%) of plantations developed between 1973 and 2015 (9.1 Mha) were established on land that had lacked forest for more than five years. Looking at each observation period, the proportion of industrial plantations established on land that lacked forest cover has increased steadily (Fig. 5A,B). Much of this pattern of expansion on lands cleared long before establishment is explained by oil-palm in Indonesian Borneo: 1.8 Mha (55%) of oil-palm plantations added since 2005 (3.3 Mha) were developed on land that lacked forest cover for at least five years (Figure S5a). A similar result is evident for land lacking forest cover for at least ten years prior to planting. This trend reflects the high proportion of oil-palm plantations developed on lands cleared before 1973 and on degraded lands (predominantly forests damaged by drought and recurrent burning) (Figs 2 and 3). In Malaysian Borneo, planting on lands cleared before 1973 account for less than 26% by area over the 1973–2015 period (Fig. 5C).

**Figure 6:**
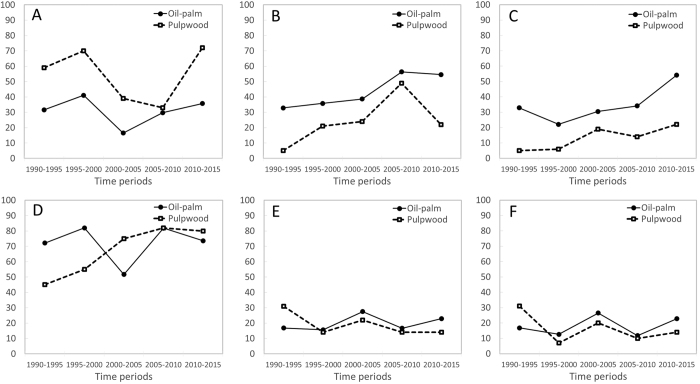
Proportion (in area terms) of industrial oil-palm (solid lines) and pulpwood (dashed lines) plantations established (**A**) on land that had forest less than five years before planting (rapid conversion), (**B**) on land that lacked forest cover for at least five years, or (**C**) for at least ten years prior to planting in Indonesian Borneo. (**D–F**) is for Malaysian Borneo.

Pulpwood plantations follow somewhat distinct patterns. Though the areas involved are smaller than for oil-palm (1.3 Mha vs 7.8 Mha), the areas converted to pulpwood plantations involve a greater proportion of rapid, within-five-year, conversion – a difference that is especially marked in Indonesia Borneo and occurs throughout the four decade study period (Fig. 6).

## Discussion

### Cause or effect?

Our study examined the rapidity with which forests (old-growth and selectively logged) have been converted into industrial plantations in Borneo over four decades. It is the first to systematically examine the time-delay between deforestation and plantation development, and to compare these losses to total deforestation across four decades over the entire island. We are thus better able to assess whether industrial plantations are the cause or consequence of forest area loss in Borneo. Unsurprisingly, they are both, though the extent and contribution of each varies by country and period.

Our study suggests that the plantation industry was the principle driver of the loss of old-growth forest in Malaysian Borneo, as 57–60% of all deforestation over four decades was associated with rapid conversion (within five years of forest clearance) to industrial plantations. In Indonesian Borneo, only 15–16% of all deforestation in Kalimantan was associated with rapid conversion to industrial plantations (11–13% attributed to oil-palm) as the majority of oil-palm plantations were developed on lands cleared before 1973 and on degraded lands (predominantly forests converted to scrublands by drought and recurrent burning during El Niño years). Despite this positive assessment Indonesian Borneo has experienced a steep increase in rapid within-five-year conversion since 2005, and became the principle contributor of rapid net forest conversion by area (2.2 Mha converted from 2005 to 2015 in Borneo, with 1.2 Mha in Indonesian Borneo). Our analysis also reveals a higher proportion of rapid forest conversion to pulpwood plantations compared with oil-palm in Indonesian Borneo. One important difference between Indonesian pulpwood and palm-oil is that the former industry has been reliant on wood from natural forests before establishing plantations^44^. This likely reflects the simpler conversion of one forest use to another within state forest land (from Production Forest to Conversion Forest) rather than the formal excision of land from the national Forest Estate required for oil-palm development. Despite the differences between the two industries in land use change, they have in common that rapid conversion of forest to industrial plantations represents a growing cause of deforestation in Indonesian Borneo.

### Caveats

We have already highlighted that our five-year conversion criteria provides a simple means to characterise broad scale patterns, and is not intended as a means to attribute blame in individual cases. Many of the patterns and differences we observe persist with longer intervals. The benefit of our approach is that it provides objective data on a contested problem comparable across countries and crop type (oil-palm and pulpwood). Given the conflicting views on this subject we highlight that while our broad-scale results appear robust there will be uncertainties in their interpretation, with some of which relating to our data.

Our data relate to land converted to large-scale industrial plantations as seen in satellite imagery (LANDSAT). These should not be confused with the total areas impacted by plantation developments. Small-holder plantations cannot consistently be distinguished in our imagery. In Indonesian Borneo, in 2013, industrial oil-palm plantations represented 89% of all oil-palm by area according to official statistics^5^ suggesting 11% of small-holder plantings, some of which is likely to have replaced old-growth forest. Our study also neglects roads, processing plants, and other infrastructure associated with plantation industries. It also neglects the impacts of the immigrant labour forces and the developments and activities that they bring and that grow around them. For example, oil-palm requires one worker for each 4–6 ha, depending on maturity of the plantings, and the 1.3–2 million workers in Borneo’s 8.0 Mha of industrial oil-palm likely require access to an additional several hundred thousand ha of land, indicating that the ultimate impacts on old-growth forests is greater than those due to planting alone. In addition, our study did not look at forest fires in relation to plantation development, nor did we estimate the impacts of the oil-palm industry on secondary forests: regrowth, forest fallow, and agro-forests. We recognize that some plantations developed on lands cleared before 1973 may have replaced agro-forests^40^,^45^. Further research is required to quantify these wider impacts.

There are cases where oil-palm developers were responsible for forest clearance even though no plantations resulted. New plantations have sometimes been approved, and the land subsequently cleared, only for the investor to withdraw without the plantation being planted^46^. Such investor withdrawal can be due to legitimate problems, though it can also result from a profitable form of fraud that generates revenues from the timber derived from forest clearance before the investor disappears^46^,^47^. We lack estimates for the areas involved. Investors or companies acquire land and hold it for extended periods before developing plantations – when such practices involve land clearance long before planting our analyses would fail to indicate the role of plantations in motivating the conversion. The acquisition of suitable land is known to improve company value and borrowing capacity which certainly encourages land banking^27^ though profits are likely to be improved by rapid, rather than delayed, planting after land clearing. Though this requires further evaluation we believe such banking-with-clearance behaviours are uncommon – we have not observed such practices, and they are seldom reported in the published literature (see, e.g. refs 48,49) and have not been quantified.

The situation with the pulpwood industry is different. This fast growing industry has long outstripped plantation production and has been reliant on wood from natural forests^44^. Between 2000 and 2010, about half of the raw material consumed by pulp mills came from the clearing of natural forest^28^, mainly on Sumatra island. In 2010, only about half of the area allocated for timber plantations (4.9 million out of more than 10 million hectares) had been planted. This means that this industry may generate significant forest loss with delayed plantation development – a process that would not be detected in our study.

There are many debates surrounding plantations and their environmental, economic and social impacts^2^,^28^,^34^,^35^. Here we are addressing only one element in these debates by improving knowledge on the rate at which natural forest is converted to plantations. Our study says nothing concerning the longer-term viability of the forests or the plantations, and the goods and services that they provide.

### National differences

Indonesia, Malaysia and Brunei are subject to different histories, regulatory systems and land-use outcomes^18^,^50^. One recent review examines and contrasts the contexts, policies and intent of oil-palm developments in Indonesia and Malaysia though they also highlight some recent convergence^27^ – and the interested reader should examine that more detailed and comprehensive account – here we highlight only some key factors.

The expansion of the plantation sector is determined by similar opportunities in both Indonesia and Malaysia. Availability of land and labour, and for oil-palm at least, high production potential, good markets and high profitability. Considerable global growth in demand for palm-oil and pulpwood is anticipated. Plantations are required to meet this demand. Plantation companies and their shareholders, which include governments, seek to maximise the marginal returns to their capital and seek access to large expanses of cheap, unencumbered land with access to reliable low-cost labour. The migratory labourers in the Indonesian Workforce (the *Tenaga Kerja Indonesia* or TKI) have sustained the sector in both Malaysia and Indonesia though there is some diversification in Malaysia in recent years. Neither country has enforced their land-use plans: both have repeatedly modified their “permanent forest estate” to allow expansion of plantations. As the most accessible sites have been planted attention has increasingly focused on attracting investments to more remote and sparsely populated areas such as Borneo^27^.

According to the Indonesian constitution forest lands are under state control, and previous governments sought to impose state ownership, but the laws governing Indonesia’s forest lands remain in a state of flux, subjected to national, regional, district, and traditional claims^51^,^52^. This has led to a complex system in which forest can be converted in multiple ways by multiple actors with contrasting and sometimes overlapping authority. This complexity has contributed to an often unclear situation in terms of who has what rights to forests and forest lands^53^ (we acknowledge that the current government shares this concern and is taking steps to address it with the One Map Policy, whose remit is to identify and resolve overlapping land claims in rural areas^54^. The recent increase in forest conversion is likely associated with Indonesia’s political decentralization that followed 1998 and the fall of President Suharto. This resulted in increased local claims over the national forest estate, providing new opportunities for plantation development on forest lands. Initially informal land claims can be used to obtain local level permits to plant oil-palm even when national-level authorisation, or release of land from the forest estate, remains pending. In Malaysia, by contrast national and state-level government top-down planning seems to drive spatial planning for plantations. Long-term land title/leases are recognised and have been expanded into forest land to encourage commercial plantations^50^. In such a formal and regulated context, the process of allocating and developing forest for oil-palm has been more consistent and systematic and likely somewhat less prone to delays. Another major difference between Indonesian and Malaysian Borneo is the much greater availability of degraded lands in the former reducing the need for forest conversion in order to develop plantations. Brunei provides a third example, highlighting how a country with considerable oil-wealth, and a limited labour force, is less likely to commit to a land and labour intensive activity such as palm-oil production^55^.

### Commitments, regulations and transparency regarding oil-palm

Recent debate on plantation development focus on oil-palm so we shall now focus on what our study reveals in that context. The Roundtable on Sustainable Palm Oil (RSPO), as the dominant certification system for palm-oil, plays a key role in defining good practice and fulfilling consumer expectations as the oil-palm industry continues its global expansion^56^,^57^. To comply with current RSPO standards requires clear evidence of prior land clearance before plantation development using reference dates^58^. After these dates, companies causing forest loss cannot be certified unless adequate compensations are made (financial or set-aside lands). The RSPO lacks information about land-clearing trajectories for many of its members and assessments are made on a case-by-case basis. Our approach offers an objective (if imperfect) method to identify where deforestation criteria appear to be violated. Our assumptions would not be necessary if the oil-palm business itself took sufficient steps towards transparency and accountability. Such steps would be in accord with the calls for greater transparency in general which are increasingly heard from within the industry as well as from others^59^.

In addition to certification, many palm-oil companies are making zero-(net) deforestation commitments to placate consumer concerns and overcome import restrictions in Europe and elsewhere^60^. A clear, practical and agreed definition of what this commitment means, is lacking. The period between forest loss and plantation development could be one useful criterion. Technically it should not be hard to develop an online system that shows land cover history and current land use and ownership. Such systems would improve transparency and accountability and would greatly aid organizations such as RSPO and “zero-deforestation” commitments to generate the credibility they desire. The timing of forest loss and plantation establishment would be crucial data. Improved methods could incorporate analyses like ours, but enhanced by the active involvement of companies themselves by committing to making their concession boundaries, and histories of their plantations available. Implementation of these measures would give both buyers and sellers of palm-oil greater clarity about good and bad practices.

## Conclusion

Our study helps break the dichotomy between opponents and proponents of oil-palm by clarifying that the role of oil-palm in deforestation is dependent not on the plant but on where it is planted. Given the conflicting claims and concerns surrounding plantations, any objective information offers new insights. Several patterns revealed by our analyses are robust. Borneo, Indonesian Borneo in particular, suffered large-scale forest loss prior to the expansion of Industrial plantations. This cleared land permitted the development of some large-scale industrial plantations without necessarily causing additional forest loss. Nonetheless, in the last decade plantations have become the primary cause of direct deforestation. Finally, we underline that the debate concerning which plantation developments are benign and which are harmful is likely to continue until the ownership and regulatory history of plantation lands is opened to wider scrutiny.

## Methods

### Borneo-wide deforestation

We mapped the Borneo-wide decline in forest area over four consecutive periods (1973–2000, 2000–2005, 2005–2010, 2010–2015) by combining four published LANDSAT-based datasets^4^,^7^,^61^,^62^.

First, we combined two comparable *Forest* maps^4^,^7^, each showing the extent of natural forests for year 1973 and year 2000 to determine the decline in forest area for the period 1973–2000. Both maps were derived using supervised (semi-automatic) computer codes that analyzed the spectral colors of *Forest* and *Non-Forest* on LANDSAT imagery. For both datasets, the definition of *Forest* includes any natural closed-canopy evergreen forest that has remained in sufficiently good condition to be classified as intact or nearly intact forest in the near-infrared, mid-infrared, and red bands of LANDSAT imagery (see Figure S6). This definition includes old-growth forest (Dipterocarps and Kerangas on dry mineral soils, on fresh-water and peat swamps as well as mangrove forests), selectively logged forest, and some forest only mildly impacted by ground fires^7^. It excludes young forest regrowth, scrublands (see paragraph on scrublands below), tree plantations, agricultural land, and non-vegetated areas. Areas classified as *Forest* in 1973 and *Non-Forest* in 2000, were recoded as *Deforestation* for the period 1973–2000. Areas classified as Non-*Forest* in 1973 and *Forest* in 2000, were recoded as *Forest* from 2000 and onwards (977,770 ha).

Second, we determined the decline in forest area for recent periods (2000–2005, 2005–2010, and 2010–2015) by combining the 2000 *Forest* map with a *Tree Loss* map, derived using an automatic computer code, and revealing annual losses of trees from January 2001 to December 2015^61^,^62^. We highlight that this *Tree Loss* map does not distinguish between removal of natural and planted trees^4^. To reduce any error resulting from this ambiguity, we excluded *Tree Loss* pixels outside of the area occupied by natural forests according to the 2000 *Forest* map, to only determine losses in natural forest area rather than losses in planted trees. In doing so, we generated three *Deforestation* maps for 2000–2005, 2005–2010, and 2010–2015 and three additional *Forest* maps for years 2005, 2010 and 2015. The final Borneo-wide map is a map showing losses in natural forest area over four consecutive periods between 1973 and 2015.

### Mapping the expansion of industrial plantations

The *Deforestation* map described above does not determine the cause and purpose of forest loss. To investigate the role of industrial plantations in deforestation, we combined the *Deforestation* map with *a* map showing the expansion of areas developed by companies, created from a time-series of 434 LANDSAT images (Table S6) arranged in sequence in *circa* 1973, 1990, 1995, 2000, 2005, 2010, and 2015.

First, we declared an area *Developed-by-Company* (the land is either already planted or under development), the moment we observed large rectangular elements, long linear boundaries, and distinctive grid- or contour-planting patterns appear on our sequence of images. These planting patterns characterize industrial plantations (Figure S7). They are easily detected by the human eye, but are difficult to capture with computer codes. *Object-Oriented* codes cannot separate between the great mixtures of land parcels arrangements. *Spectral-Color* algorithms cannot distinguish closed-canopy oil-palm plantations from other closed-canopy vegetation types, or open-canopy plantations from bare lands. Therefore, we delineated the boundaries of the areas developed (or under development) by companies using a visual, expert-based interpretation method. We also employed maps of oil-palm and pulpwood concessions that have entered the public sphere^41^ to distinguish young oil-palm from young pulpwood plantations because similar planting patterns and spectral colors are seen in both plantation types. In the process, we also generated the expanding area of pulpwood plantations. We note, that concession maps were not always a reliable indicator of plantations locations. Particularly for Indonesia, the oil-palm industry often develops plantations outside of concession boundaries including inside pulpwood concessions^40^. The opposite is not true because pulpwood plantations are better regulated. A total of 1,705 and 366 polygons (>90 ha) developed by oil-palm and pulpwood companies, respectively, each with an associated year of development, were digitized in ArcGIS 10.2.2, by just two experts working together, in the same office, to ensure consistency.

Second, we corrected the *Developed-by-Company* polygons to remove any obvious errors resulting from visual interpretation. Small remnants of remaining forests or small patches of undeveloped clearings (for example, openings in the forest by small-holders, or logging tracts) were counted as *Developed-by-Company* during visual interpretation. It is often too time-consuming to digitize small elements manually, but these small elements were detected by the computer codes used to generate the *Deforestation* map. We refined the shape (and size) of the *Developed-by-Company* polygons by intersecting them with the *Deforestation* map, which reveal these discrepancies between both datasets. For example, if an area was *Forest* in 2015 according to the *Deforestation* map, and *Developed-by-Company* according to the visual interpretation, the area was recoded as *Forest* (see example in Figure S8). Conversely, if an area was *Cleared* according to the *Deforestation* map, and *Forest* according to visual interpretation, we determined the correct land cover type (whether *Cleared*, or *Developed*) by reviewing our database of LANDSAT imagery and, in some cases high-resolution imagery on Google Earth. The same procedure was repeated for previous years. This correction procedure ensured total consistency between the Borneo-wide *Deforestation* map and the map showing the expansion of areas developed by companies.

Third, for each corrected *Developed-by-Company* polygon, we determined in which previous periods *Forest* was present and then *Cleared/Deforested*, again by intersecting them with the Borneo-wide *Deforestation* map. For the two target years (1990, 1995), for which no island-wide *Deforestation* map existed, we delineated the *Forest* boundary inside each *Planted* polygon by visual interpretation (Figure S9). We declared an area *Forest* when we identified spectral colors characteristic of old-growth or selectively logged closed-canopy evergreen forests in Near-Infrared, Mid-Infrared, and Red bands. We declared an area *Cleared* when the presence of forest was no longer evident following conversion to small-scale agriculture, mining, permanent flooding, drought and fire.

Fourth, we calculated the time that elapsed between deforestation and planting by taking the time difference between the year we declared the land *Cleared/Deforested* and the year we declared it *Planted*. The minimum time-delay was five years because our time-series was arranged in five-year increments. We defined *Rapid Conversion* areas where we observed planting to occur within five years of clearance.

Fifth, areas obscured by clouds or where imagery was missing, were marked as *Uncertain*. If the imagery acquired prior to and following a cloudy image (or no data), indicated no change in land cover, the *Uncertain*’ category was recoded to the corresponding land cover. To reduce the area of uncertainty, we also inspected four Borneo-wide cloud-free LANDSAT mosaics for years 2000, 2005, 2013 and 2014^61^,^63^ as well as radar imagery for year 2010^64^.

Finally, we compared our *Plantation* map with two previously published maps of planted industrial oil-palm over Indonesian Borneo (Kalimantan), also created by visual interpretation and from 1990 to 2010.

### Map validation

We checked the accuracy of our *Forest*, *Non-Forest*, and *Plantation* maps by following good practice guidelines^65^,^66^. We used Google Earth’s high-resolution images (0.6–1 m resolution; range of years: 2000–2015) as a reference dataset for five independent validations. These comprised four single-date accuracy assessments to validate the 2000, 2005, 2010 and 2015 plantation maps, and one two-date assessment to validate the plantation change map from 2010 to 2015. The reference areas of the 2000, 2005, 2010, 2015, and 2010–2015 assessments were 229,082 ha, 1,105,985 ha, 4,468,302 ha, 1,122,018 ha, respectively (Figure S10). We visually interpreted 60 m x 60 m sample areas (equivalent to four LANDSAT TM, ETM pixels) randomly distributed across the reference areas, with 265, 287, 258, 256, 429 samples used in the 2000, 2005, 2010, 2015, and 2010–2015 assessments, respectively. Each sample area was assigned either: *Plantation*, *Forest* or *Non-Forest* (single date), or: *Forest to Plantation*, *Non-Forest to Plantation*, *Plantation* to *Plantation* (two-date). We then determined the frequency of class agreement and disagreement in the samples, the actual error matrix, bias-corrected error estimates, true area estimates, and confidence intervals. These validation results are presented in supplementary results. None of Google Earth’s high-resolution images used to validate our maps are shown in this study.

### Area of forest that has been logged before being converted to oil-palm plantations

We further divided ‘forest’ into ‘intact’ and ‘logged’ using our published dataset of logging roads and a buffer distance method^7^. Briefly, we consider that the forest has never been logged if our database of images never detected the presence of large (>10 m wide) logging roads in the forest. Degradation by logging usually becomes undetectable within a few years, due to fast forest re-growth^67^. From a satellite perspective, the spectral colors of logged forests resemble those of intact forests, which explains why recently published state-of-the-art deforestation analyses categorized logged forests as forest^4^,^7^,^61^.

### Estimating the area of forest that has turned to scrublands by fire before being converted to oil-palm plantations

Burned forests appear distinct from intact and logged forests on LANDSAT imagery. These distinct spectral signals remain visible for several years^68^ (Figure S11), which explains why recently published state-of-the-art deforestation analyses detected forests severely degraded by fire as deforested^4^,^7^,^61^. To estimate the area of burned forest where scrublands have replaced forests before being converted to oil-palm plantations, we reviewed available literature to determine the timing and broad location of fires^9^,^11^,^12^,^14^,^16^,^69^,^70^,^71^,^72^,^73^,^74^. Next, we organized our LANDSAT database into imagery acquired before and after seven major fire events noted in our literature search. We then searched for changes in the spectral reflectance of forests that were consistent with changes from high tree cover and species-rich forests to a homogeneous low vegetation cover on the pre- and post-fire imagery (Figure S5). Due to the variety of forest types as well as variable image acquisition conditions in Borneo, and the persistence of cloud cover even during the dry season, we performed this assessment manually by visual interpretation. We note that scrublands mapped in this study may show regeneration potentials in some region, which we do not address in this study

### Analyses of geospatial data

All the maps presented in this article, and geospatial analyses performed in this study were carried out by the authors of this study using *ArcMap* v10.0 geospatial processing program.

## Additional Information

**How to cite this article**: Gaveau, D. L. A. *et al*. Rapid conversions and avoided deforestation: examining four decades of industrial plantation expansion in Borneo. *Sci. Rep*. **6**, 32017; doi: 10.1038/srep32017 (2016).

## Supplementary Material

Supplementary Information

## Figures and Tables

**Table 1:** Forest and industrial plantations (oil-palm and pulpwood combined) area and change by country.

Areas (in Ha)	Borneo	Kalimantan	Sabah	Sarawak	Brunei
Total land area	73,719,011	53,342,225	7,396,621	12,400,501	579,664
Forest area in 1973	55,836,571	40,325,220	5,833,479	9,224,349	453,523
Non forest area in 1973	16,349,575	11,918,546	1,233,951	3,090,837	106,241
Forest area loss (Deforestation):					
1973–2000	13,933,352	11,489,956	1,398,721	1,007,064	37,611
2000–2005	900,575	495,872	141,634	261,334	1735
2005–2010	1,969,608	1,140,535	204,522	621,352	3,199
2010–2015	1,847,258	1,228,226	117,498	497,443	4091
**Total deforestation (1973–2015)**	**18,650,793**	**14,354,589**	**1,862,375**	**2,387,193**	**46,636**
Intact forest area in 2015	20,531,822	16,798,025	1,647,149	1,756,476	330,172
Logged forest area in 2015	16,802,893	9,323,126	2,322,139	5,080,871	76,757
**Total forest area in 2015**	**37,334,715**	**26,121,151**	**3,969,288**	**6,837,347**	**406,929**
Area of plantations in 1973	132,809	0	128,047	4,762	0
Plantations expansion:					
1973–1990	952,979	247,205	601,175	104,599	0
1990–1995	1,014,415	613,133	279,000	122,282	0
1995–2000	1,510,327	781,011	382,608	346,004	704
2000–2005	858,764	484,981	136,777	236,730	276
2005–2010	2,568,834	1,882,201	132,852	552,790	991
2010–2015	2,208,196	1,690,168	118,049	399,325	654
Total expansion (1973–2015)	9,113,515	5,698,699	1,650,461	1,761,730	2,625
Total Area of plantations in 2015	9,246,324	5,698,699	1,778,508	1,766,492	2,625
Total forest area converted to plantations within five years of clearance^*^ (1973–2015)	4,537,052	2,113,815	1,086,468	1,334,168	2,601
Total forest area cleared more than five years before plantations were established (1973–2015)	1,798,114	1,623,829	71,555	102,720	10
Total forest area cleared within five years or between five and ten years before plantations were established	282,922	211,294	38,630	32,988	10
**Total forest area converted to plantations during 1973–2015**	6,968,935	4,276,910	1,212,447	1,476,957	2,621
Total area of land lacking forest cover in 1973 and converted to plantations (1973–2015)	1,784,453	1,258,184	243,740	282,526	3
Total scrub area (i.e. forests severely degraded by fire) converted to plantations (1973–2015)	1,469,717	1,402,115	48,310	19,292	0
