# Supplementary material for: Rapid conversions and avoided deforestation: examining four decades of industrial plantation expansion in Borneo
Source: Sci Rep. 2016 Sep 8;6:32017. doi: 10.1038/srep32017 (PMC5015015; doi:10.1038/srep32017)
Supplement: Supplementary Information [file srep32017-s1.docx]

**Rapid conversions and avoided deforestation: examining four decades of industrial plantation expansion in Borneo.**

David L.A. Gaveau^1^, Douglas Sheil^2^, Husnayaen^1^, Mohammad A. Salim^1^, Sanjiwana Arjasakusuma^1^, Marc Ancrenaz^3,4^, Pablo Pacheco^1^, Erik Meijaard^,3,5^

1 Center for International Forestry Research, P.O. Box 0113 BOCBD, Bogor 16000, Indonesia

2 Department of Ecology and Natural Resource Management (INA), Norwegian University of Life Science (NMBU), Box 5003, 1432 Ås, Norway

3 Borneo Futures project, People and Nature Consulting International, Ciputat, Jakarta, 15412, Indonesia.

4 HUTAN, Kinabatangan Orang-utan Conservation Programme, Kota Kinabalu

5 School of Biological Sciences, University of Queensland, Brisbane, QLD 4072, Australia.

**Supplementary information**

**Supplementary Tables**

**Table S1a.** Industrial oil-palm plantation area and change by country.

| Areas (in Ha) | Borneo | Kalimantan | | Sabah | | Sarawak | | Brunei | |
| --- | --- | --- | --- | --- | --- | --- | --- | --- | --- |
| Total land area | 73,719,011 | 53,342,225 | 7,396,621 | | 12,400,501 | | 579,664 | |  |
|  |  |  |  | |  | |  | |  |
| Area of plantations in 1973 | 132,801 | 0 | 128,039 | | 4,762 | | 0 | |  |
| Plantations expansion: |  |  |  | |  | |  | |  |
| 1973–1990 | 834,380 | 168,084 | 561,697 | | 104,599 | | 0 | |  |
| 1990–1995 | 777,329 | 386,974 | 268,073 | | 122,282 | | 0 | |  |
| 1995–2000 | 1,197,938 | 542,471 | 335,996 | | 318,981 | | 490 | |  |
| 2000–2005 | 719,451 | 421,841 | 109,335 | | 188,274 | | 1 | |  |
| 2005–2010 | 2,356,132 | 1,777,070 | 87,564 | | 491,338 | | 160 | |  |
| 2010–2015 | 1,968,786 | 1,544,143 | 77,373 | | 347,243 | | 27 | |  |
| Total expansion (1973–2015) | **7,854,016** | **4,840,583** | **1,440,038** | | **1,572,717** | | **678** | |  |
| Total Area of plantations in 2015 | 7,986,817 | 4,840,583 | 1,568,077 | | 1,577,479 | | 678 | |  |
|  |  |  |  | |  | |  | |  |
| Total forest area converted to plantations within five years of clearance^*^ (1973–2015) | 3,711,511 | 1,577,672 | 970,506 | | 1,162,658 | | 675 | |  |
| Total forest area cleared more than five years before plantations were established (1973–2015) | 1,576,397 | 1,427,648 | 51,723 | | 97,026 | | 0 | |  |
| Total forest area cleared within five years or between five and ten years before plantations were established | 229,299 | 179,790 | 19,526 | | 29,983 | | 0 | |  |
| Total forest area converted to plantations during 1973 – 2015 | **5,817,445** | **3,473,469** | **1,048,027** | | **1,295,274** | | **675** | | |
|  |  |  |  | |  | |  | | |
| Total area of land lacking forest cover in 1973 and converted to plantations (1973–2015) | 1,689,242 | 1,207,921 | 206,124 | | 275,194 | | 3 | | |
| Total scrub area (i.e. forests severely degraded by fire) converted to plantations (1973–2015) | 1,264,676 | 1,227,916 | 18,029 | | 18,731 | | 0 | | |

**Table S1b.** Industrial pulpwood plantation area and change by country.

| Areas (in Ha) | Borneo | Kalimantan | | Sabah | | Sarawak | | Brunei | |
| --- | --- | --- | --- | --- | --- | --- | --- | --- | --- |
| Total land area | 73,719,011 | 53,342,225 | 7,396,621 | | 12,400,501 | | 579,664 | |  |
|  |  |  |  | |  | |  | |  |
| Area of plantations in 1973 | 9 | 0 | 9 | | 0 | | 0 | |  |
| Plantations expansion: |  |  |  | |  | |  | |  |
| 1973–1990 | 118,600 | 79,121 | 39,479 | | 0 | | 0 | |  |
| 1990–1995 | 237,085 | 226,159 | 10,926 | | 0 | | 0 | |  |
| 1995–2000 | 312,389 | 238,540 | 46,612 | | 27,023 | | 214 | |  |
| 2000–2005 | 139,315 | 63,140 | 27,443 | | 48,457 | | 275 | |  |
| 2005–2010 | 212,702 | 105,131 | 45,288 | | 61,453 | | 830 | |  |
| 2010–2015 | 239,413 | 146,026 | 40,678 | | 52,082 | | 627 | |  |
| Total expansion (1973–2015) | **1,259,504** | **858,117** | **210,426** | | **189,015** | | **1,946** | |  |
| Total Area of plantations in 2015 | **1,259,513** | **858,117** | **210,435** | | **189,015** | | **1,946** | |  |
|  |  |  |  | |  | |  | |  |
| Total forest area converted to plantations within five years of clearance^*^ (1973–2015) | 825,545 | 536,144 | 115,963 | | 171,511 | | 1,927 | |  |
| Total forest area cleared more than five years before plantations were established (1973–2015) | 221,719 | 196,183 | 19,832 | | 5,694 | | 10 | |  |
| Total forest area cleared within five years or between five and ten years before plantations were established | 53,623 | 31,504 | 19,104 | | 3,005 | | 10 | |  |
| Total forest area converted to plantations during 1973 – 2015 | **1,151,490** | **803,441** | **164,420** | | **181,683** | | **1,946** | | |
|  |  |  |  | |  | |  | | |
| Total area of land lacking forest cover in 1973 and converted to plantations (1973–2015) | 95,211 | 50,263 | 37,616 | | 7,332 | | 0 | | |
| Total scrub area (i.e. forests severely degraded by fire) converted to plantations (1973–2015) | 205,041 | 174,199 | 30,281 | | 561 | | 0 | | |

**Table S2. Results of single-date assessment to validate the *Forest*, *Non-Forest*, and *Plantation* maps for year 2000.** Error matrix with estimated area and user, producer and overall accuracy with the confidence interval.

|  | |  | | Reference | | | Area (ha) | Estimated Area (ha) | CI Area |
| --- | --- | --- | --- | --- | --- | --- | --- | --- | --- |
|  |  | Planted | Forest | | Non-Forest | Total Sample |  |  |  |
| Map | Planted | 82 | 8 | | 42 | 132 | 1,351 | 839 | 112 |
|  | Forest | 0 | 71 | | 2 | 73 | 122,996 | 130,302 | 9342 |
|  | Non-Forest | 0 | 6 | | 54 | 60 | 105,940 | 99,146 | 9342 |
|  | Total Sample | 82 | 85 | | 98 | 265 | 229,082 |  | |

| Class | User Accuracy | Producers Accuracy | CI of Users | CI of Producers | Overall Accuracy | CI |
| --- | --- | --- | --- | --- | --- | --- |
| Planted | 0.62 | 1.00 | 0.08 | 0.00 | 0.94 | 0.04 |
| Forest | 0.97 | 0.92 | 0.04 | 0.057 |  | |
| Non-Forest | 0.90 | 0.96 | 0.08 | 0.045 |  | |

**Table S3. Results of single-date assessment to validate the *Forest*, *Non-Forest*, and *Plantation* maps for year 2005.** Error matrix with estimated area and user, producer and overall accuracy with the confidence interval.

|  | |  | | Reference | | | Area (ha) | Estimated Area (ha) | CI Area |
| --- | --- | --- | --- | --- | --- | --- | --- | --- | --- |
|  |  | Planted | Forest | | Non-Forest | Total Sample |  |  |  |
| Map | Planted | 126 | 1 | | 15 | 142 | 244,925 | 229,409 | 20,994 |
|  | Forest | 0 | 47 | | 3 | 50 | 291,780 | 312,242 | 34,411 |
|  | Non-Forest | 2 | 6 | | 87 | 95 | 573,849 | 568,904 | 37,607 |
|  | Total Sample | 128 | 54 | | 105 | 287 |  |  | |

| Class | User Accuracy | Producers Accuracy | CI of Users | CI of Producers | Overall Accuracy | CI |
| --- | --- | --- | --- | --- | --- | --- |
| Planted | 0.89 | 0.95 | 0.05 | 0.07 | 0.92 | 0.04 |
| Forest | 0.94 | 0.88 | 0.07 | 0.08 |  | |
| Non-Forest | 0.92 | 0.92 | 0.06 | 0.04 |  | |

**Table S4. Results of single-date assessment to validate the *Forest*, *Non-Forest*, and *Plantation* maps for year 2010.** Error matrix with estimated area and user, producer and overall accuracy with the confidence interval.

|  | |  | | Reference | | | Area (ha) | Estimated Area (ha) | CI Area |
| --- | --- | --- | --- | --- | --- | --- | --- | --- | --- |
|  |  | Planted | Forest | | Non-Forest | Total Sample |  |  |  |
| Map | Planted | 109 | 0 | | 16 | 125 | 501,248 | 437,088 | 29,475 |
|  | Forest | 0 | 49 | | 3 | 52 | 1,729,375 | 1,656,035 | 122,192 |
|  | Non-Forest | 0 | 1 | | 80 | 81 | 2,140,980 | 2,278,480 | 122,192 |
|  | Total Sample | 109 | 50 | | 99 | 258 |  |  | |

| Class | User Accuracy | Producers Accuracy | CI of Users | CI of Producers | Overall Accuracy | CI |
| --- | --- | --- | --- | --- | --- | --- |
| Planted | 0.87 | 1.00 | 0.06 | 0.00 | 0.96 | 0.03 |
| Forest | 0.94 | 0.98 | 0.06 | 0.031 |  | |
| Non-Forest | 0.99 | 0.93 | 0.02 | 0.047 |  | |

**Table S5. Results of single-date assessment to validate the *Forest*, *Non-Forest*, and *Plantation* maps for year 2015.** Error matrix with estimated area and user, producer and overall accuracy with the confidence interval.

|  | |  | | Reference | | | Area (ha) | Estimated Area (ha) | CI Area |
| --- | --- | --- | --- | --- | --- | --- | --- | --- | --- |
|  |  | Planted | Forest | | Non-Forest | Total Sample |  |  |  |
| Map | Planted | 101 | 0 | | 2 | 103 | 1,217,625 | 1,498,762 | 335,527 |
|  | Forest | 0 | 12 | | 2 | 14 | 1,677,962 | 1,641,440 | 422,731 |
|  | Non-Forest | 3 | 2 | | 28 | 33 | 3,352,582 | 3,107,967 | 525,749 |
|  | Total Sample | 104 | 14 | | 32 | 150 | 6,248,169 |  | |

| Class | User Accuracy | Producers Accuracy | CI of Users | CI of Producers | Overall Accuracy | CI |
| --- | --- | --- | --- | --- | --- | --- |
| Planted | 0.98 | 0.80 | 0.03 | 0.178 | 0.88 | 0.08 |
| Forest | 0.86 | 0.88 | 0.19 | 0.150 |  | |
| Non-Forest | 0.85 | 0.92 | 0.12 | 0.095 |  | |

**Table S6. Results of two-date assessment to validate the change map from 2010 to 2015.** F-F: *Forest* to *Forest*; F-P: Forest to Plantation; P-P: plantation to plantation; NF-P: non-forest to plantation; NF-NF: non-forest to non-forest. Error matrix with estimated area and user, producer and overall accuracy with the confidence interval.

|  | | Reference | | | | | | Area (ha) | Estimated Area (ha) | CI Area | |
| --- | --- | --- | --- | --- | --- | --- | --- | --- | --- | --- | --- |
|  |  | F-F | F-P | P-P | NF-P | NF-NF | Total Sample |  |  |  |  |
| Map | F-F | 19 | 1 | 0 | 0 | 0 | 20 | 240,579 | 257,037 | 39,097 | |
|  | F-P | 1 | 62 | 0 | 7 | 0 | 70 | 19,695 | 35,846 | 24,004 | |
|  | P-P | 0 | 7 | 108 | 2 | 3 | 120 | 99,574 | 109,832 | 26,342 | |
|  | NF-P | 0 | 2 | 5 | 53 | 2 | 62 | 17,502 | 37,394 | 25,948 | |
|  | NF-NF | 3 | 0 | 2 | 2 | 38 | 45 | 423,081 | 360,323 | 45,401 | |
|  | Total Sample | 23 | 72 | 115 | 64 | 43 | 317 | 800,433 |  | |  |

| Class | User Accuracy | Producers Accuracy | CI of Users | CI of Producers | Overall Accuracy | CI |
| --- | --- | --- | --- | --- | --- | --- |
| F-F | 0.95 | 0.89 | 0.10 | 0.108 | 0.88 | 0.06 |
| F-P | 0.89 | 0.49 | 0.08 | 0.595 |  | |
| P-P | 0.90 | 0.82 | 0.05 | 0.192 |  | |
| NF-P | 0.85 | 0.40 | 0.09 | 0.278 |  | |
| NF-NF | 0.84 | 0.99 | 0.11 | 0.008 |  | |

**Table S7. List of all LANDSAT images used in this study (n=434).** Global Land Survey (GLS) imagery are low-cloud cover imagery and terrain corrected.

**Supplementary figures**

**
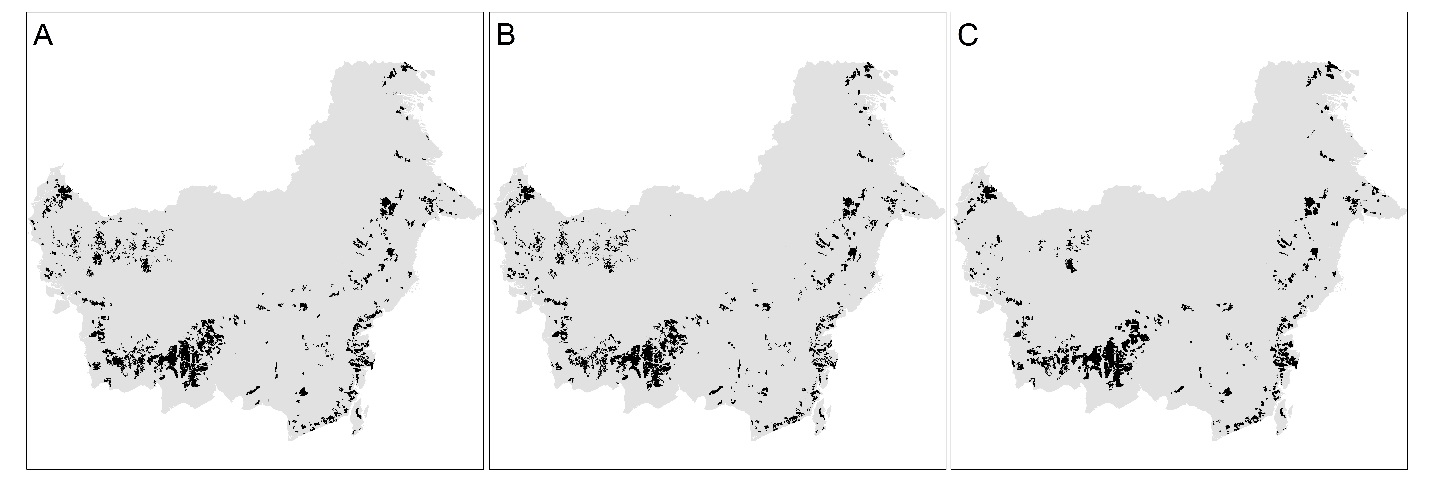
**

**Figure S1│ Comparison of industrial oil-palm plantation map created by different groups over Indonesian Borneo for year 2010.** Plantation map created by this study (A). Map adapted from Carlson et al. 2013^1^ (B). Map adapted from Gunarso et al. 2012^2^ (C). Maps created using *ArcMap* v10.2.2 geospatial processing program <http://www.esri.com/software/arcgis/arcgis-for-desktop>.


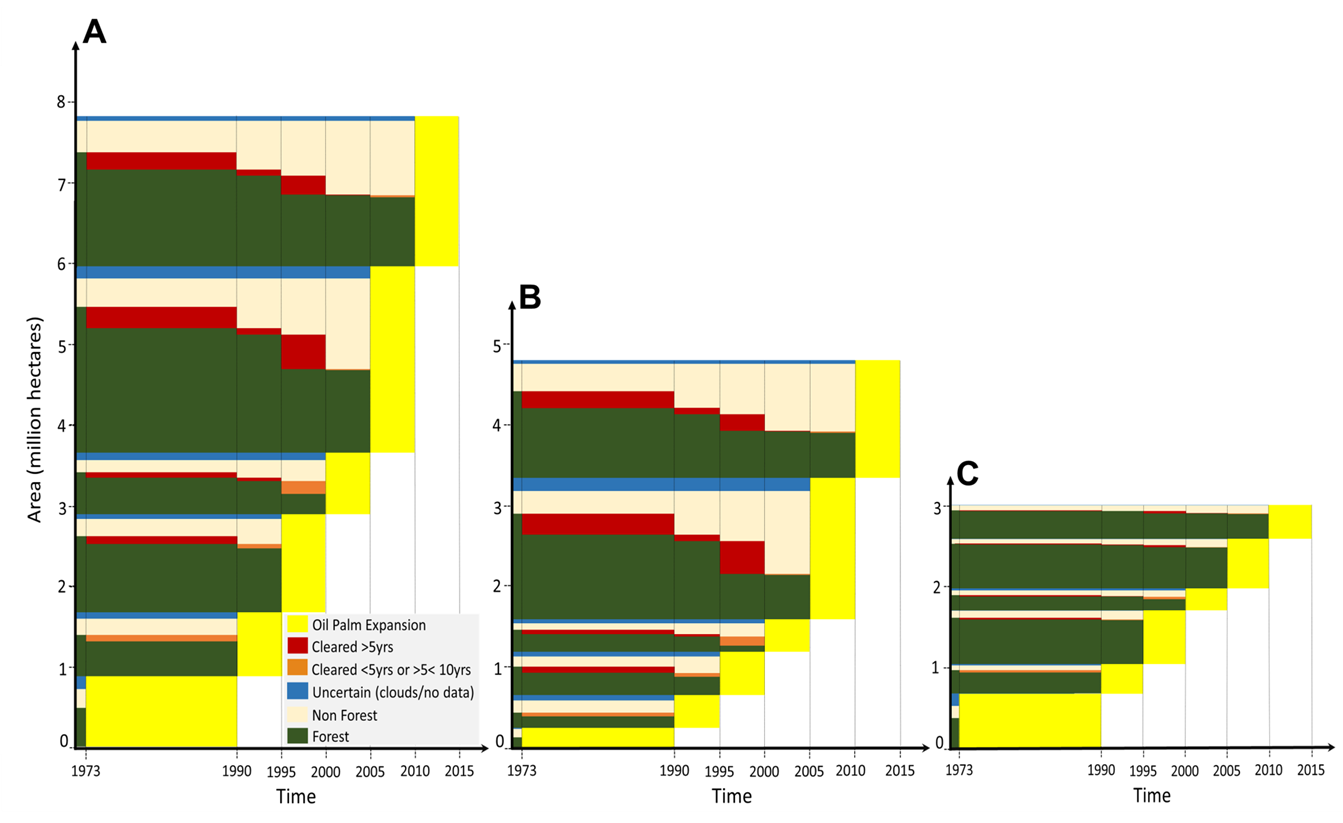


**Figure S2a│ The expanding area (7.8 Mha; yellow blocks) of industrial oil-palm plantations in six time periods from 1973 to 2015 in Borneo (A), Indonesian Borneo (B), and Malaysian Borneo (C).** The decline in forest area (dark green) since 1973, and prior to oil-palm establishment (yellow) is shown to the left of each yellow block in each time period. The changes reveal when the land was forest (green), non-forest (light brown) or had been cleared (red and orange) prior to oil-palm development (yellow). Areas cleared more than five years prior to oil-palm establishment are shown as red blocks. Areas where forest was cleared less than five years or more than five years, but less than ten years before plantation establishment are shown as orange blocks. The areas where the dark green blocks (forest) touch the yellow blocks (oil-palm) are areas where forest was cleared less than five years prior to oil-palm establishment. The blue blocks indicate areas of uncertainty, where we could not define clear land cover transitions, because of either cloud cover or lack of imagery*.*


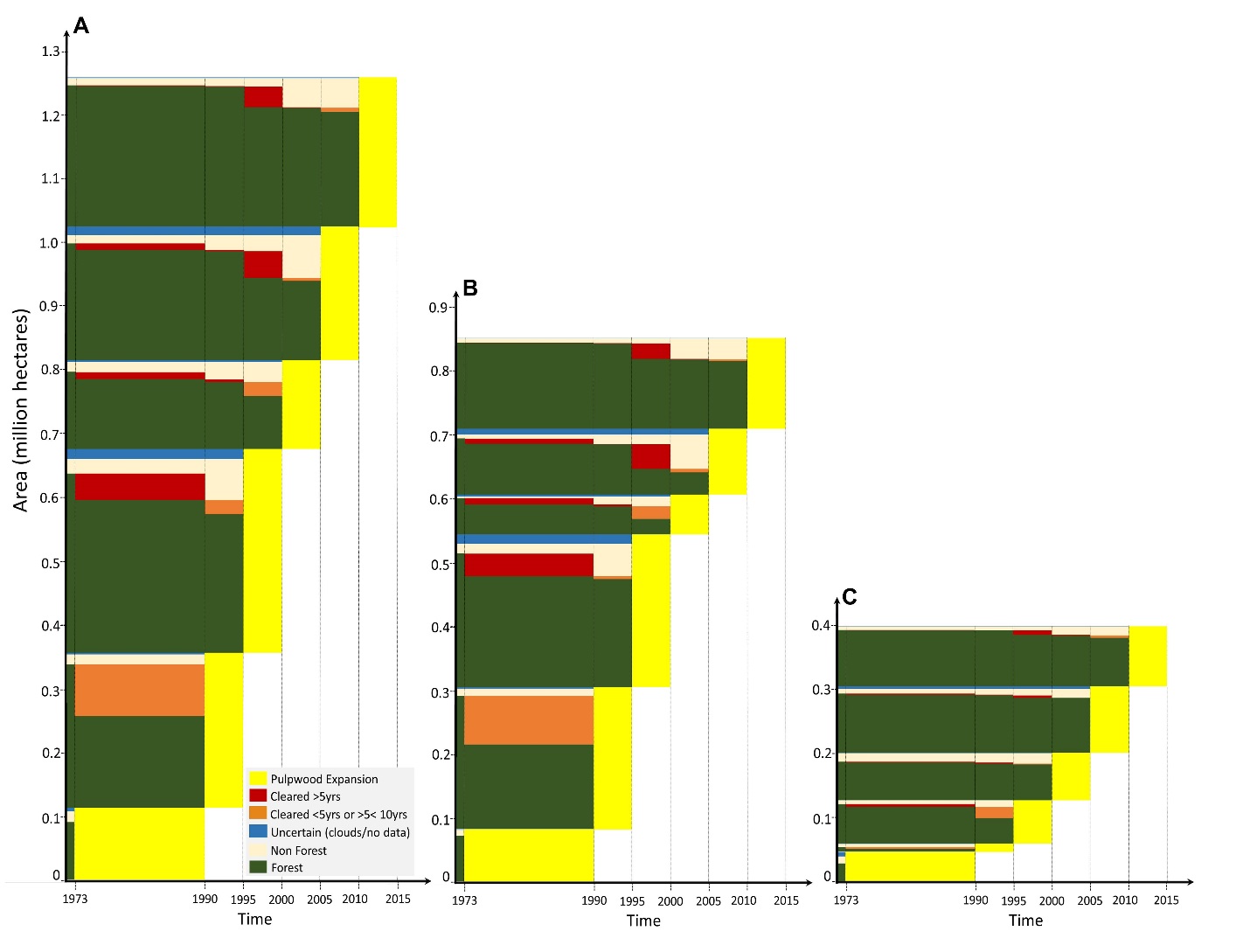


**Figure S2b│ The expanding area (1.3 Mha; yellow blocks) of industrial pulpwood plantations in six time periods from 1973 to 2015 in Borneo (A), Indonesian Borneo (B), and Malaysian Borneo (C).** For color legends refer to Figure S2a.

**
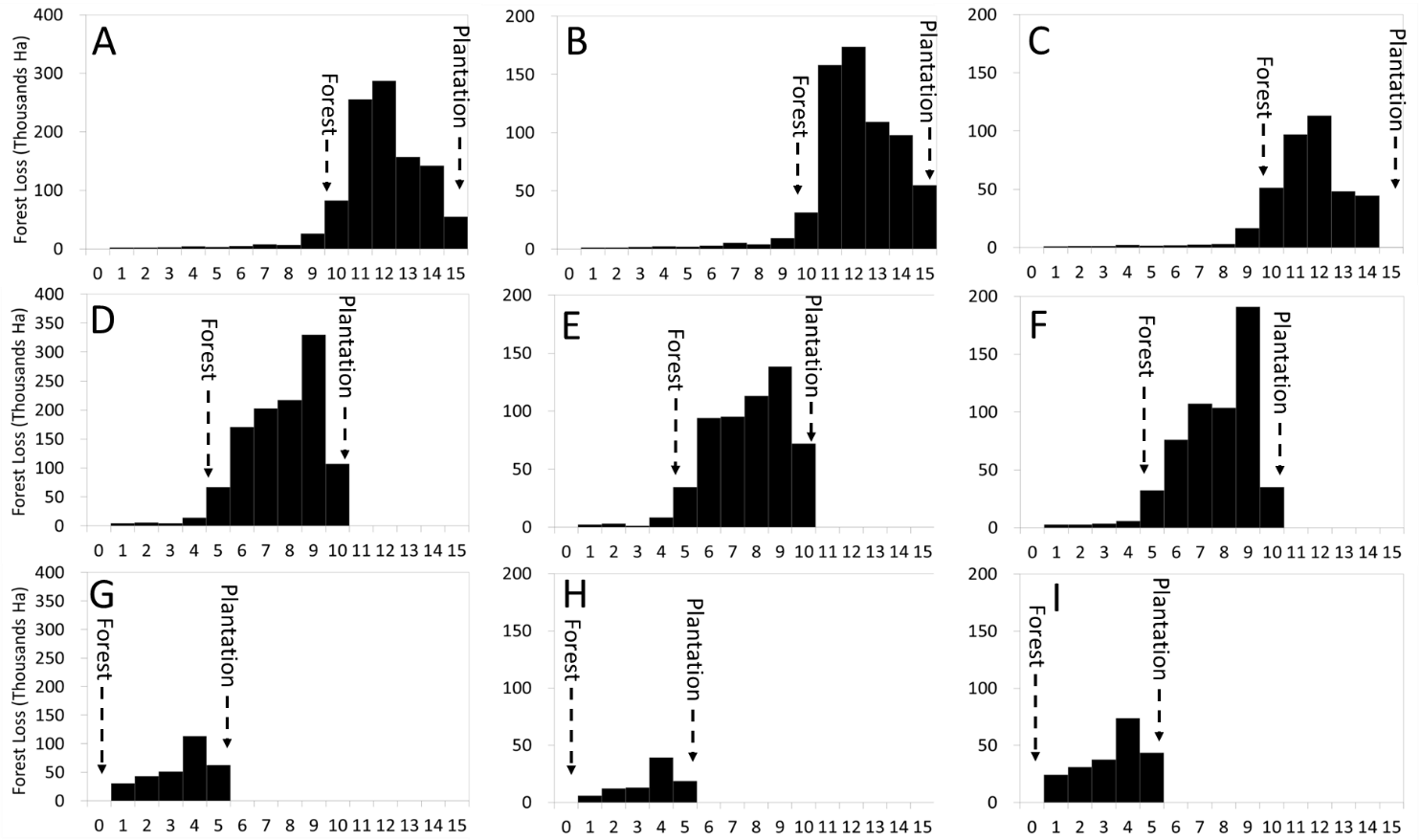
**

**Figure S2c│ Yearly variations in forest area loss from 2001 to 2015 in areas observed as having been converted to plantations (oil-palm and pulpwood) within five years of clearance** during the period 2010-2015, 2005-2010, and 2000-2005 in Borneo (A,D,G), Indonesian Borneo (B,E,H), and Malaysian Borneo (C,F,I). Here, each time interval follows a calendar year. For example, in X-axis, 1 means the area of forest lost between 01 January and 31 December 2001. The yearly data were adapted from Hansen et al.[^20^](#_ENREF_20) (See also Methods).


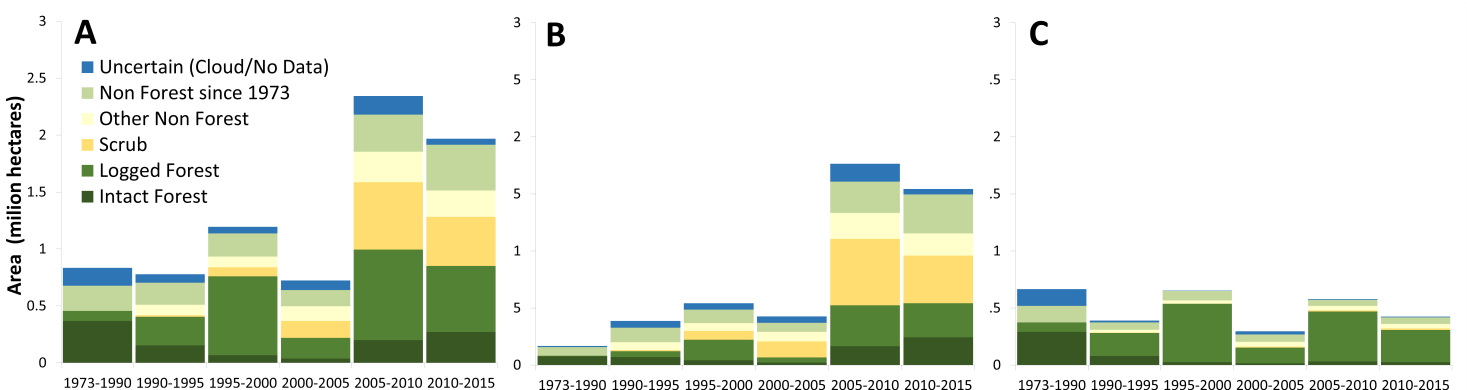


**Figure S3a│ The expanding area (7.8 Mha) of industrial oil palm plantations in six time periods from 1973 to 2015 with vegetation cover of the land just before observed conversion to oil-palm in Borneo (A), Indonesian Borneo (B), and Malaysian Borneo (C)**. **Intact Forest:** pristine old-growth forests. **Logged Forest:** old-growth forests that have lost their original structure and canopy cover through industrial-scale selective timber harvest at some point since 1973, indicated principally by the construction of logging roads. **Scrub:** old-growth forests impacted by drought and fire; these burn/drought scars tend to recover slowly. They are vulnerable to further burning and conversion to short vegetation follows; hence they appear as “deforested” in satellite assessments (see also methods). **Non Forest since 1973**: areas that have been cleared before 1973. **Other Non-Forest:** areas that have been cleared after 1973, but not converted to scrubs. We recognize that **Non Forest since 1973 and Other Non-Forest** may include secondary forests: young-growth, forest fallow or agro-forest.


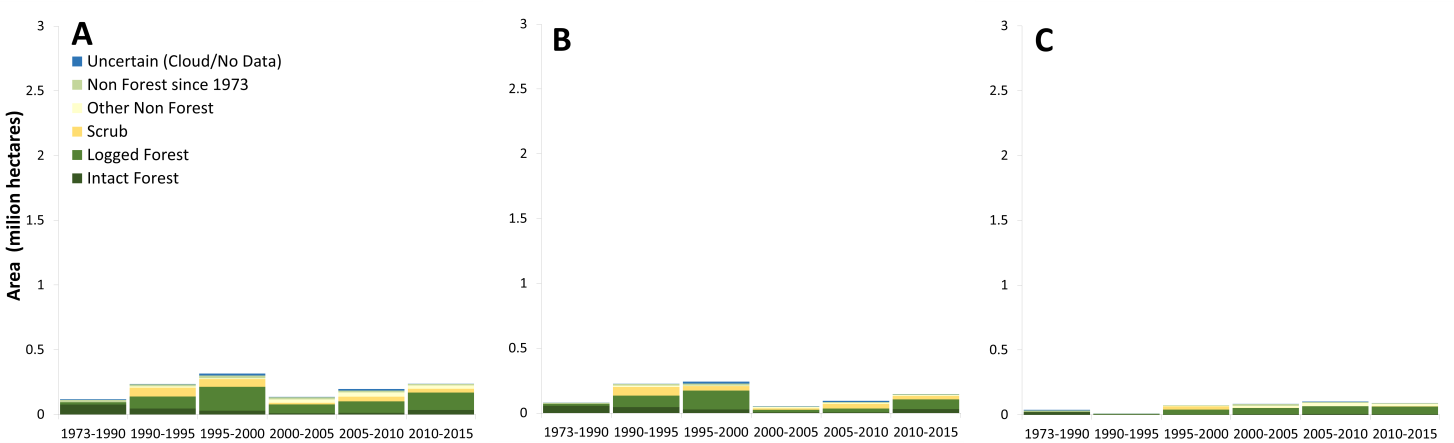


**Figure S3b│ The expanding area (1.3 Mha) of industrial pulpwood plantations in six time periods from 1973 to 2015 with vegetation cover of the land just before observed conversion to pulpwood in Borneo (A), Indonesian Borneo (B), and Malaysian Borneo (C)**.


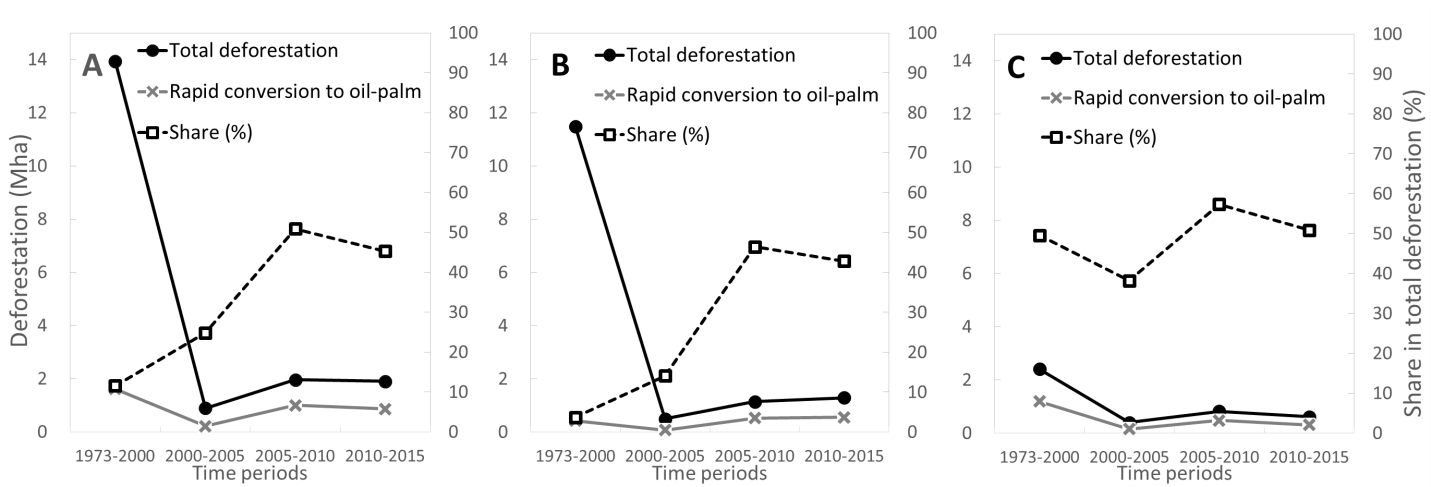


**Figure S4a│ Role of industrial oil-palm plantations in deforestation by period for Borneo (A), Indonesian Borneo (B) and Malaysian Borneo (C).** On the primary axis (left Y axis), the grey solid line indicates the area of forest rapidly converted to oil-palm (i.e. within five years of clearance), while the black solid line indicates the total area of deforestation by time period on Borneo (see also Figure 1A). On the secondary axis (right Y axis), the dashed line represents the share of rapid conversion in total deforestation, expressed in percentage terms.


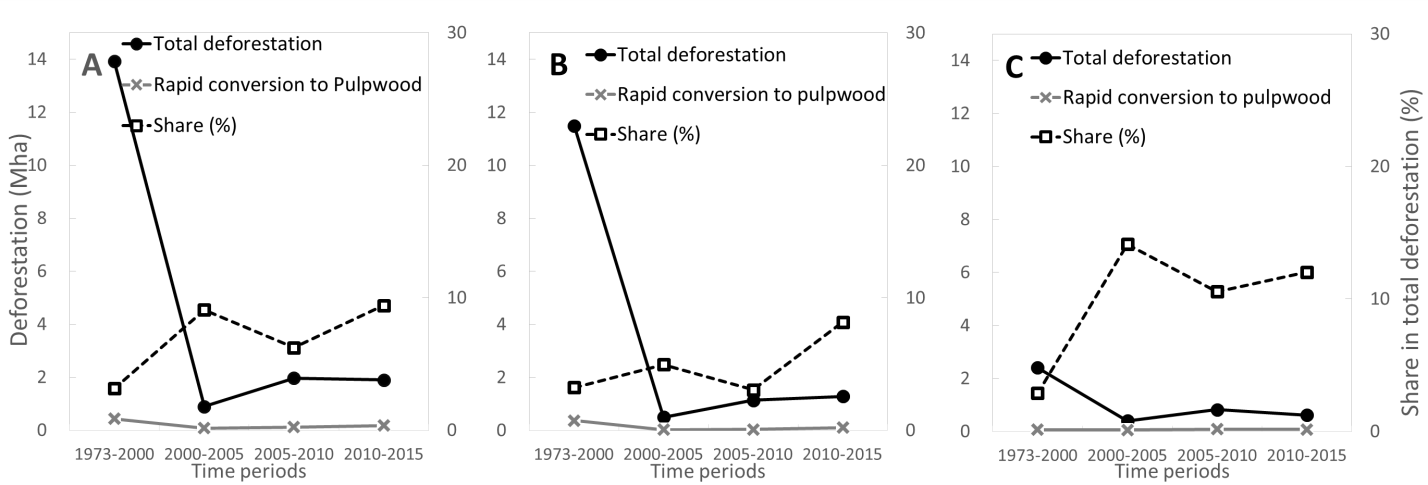


**Figure S4b│ Role of industrial pulpwood plantations in deforestation by period for Borneo (A), Indonesian Borneo (B) and Malaysian Borneo (C).** On the primary axis (left Y axis), the grey solid line indicates the area of forest rapidly converted to pulpwood plantations (i.e. within five years of clearance), while the black solid line indicates the total area of deforestation by time period on Borneo (see also Figure 1A). On the secondary axis (right Y axis), the dashed line represents the share of rapid conversion in total deforestation, expressed in percentage terms.


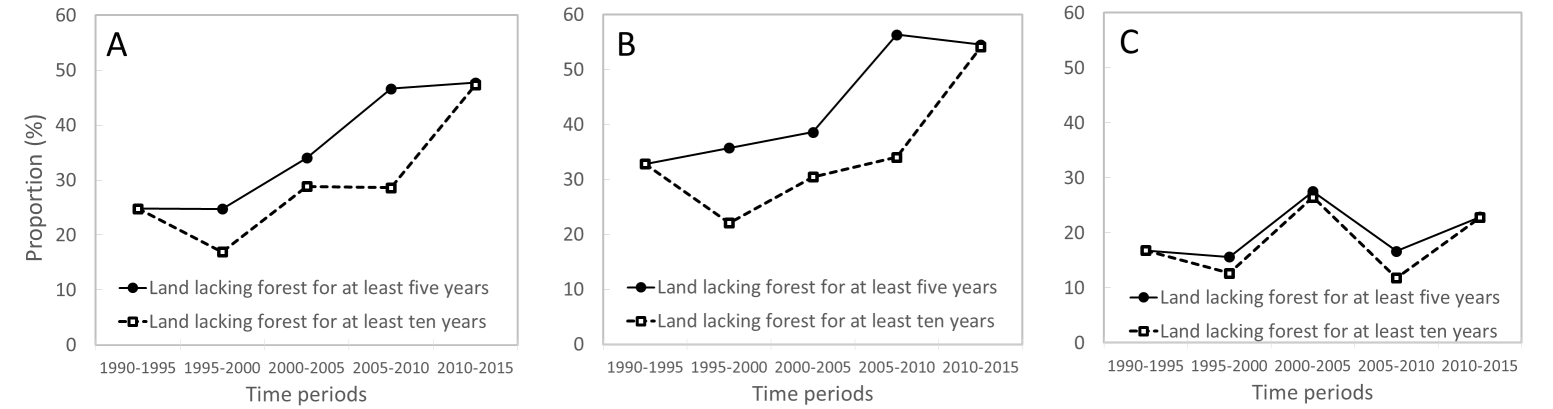


**Figure S5a│ Proportion (in area terms) of industrial oil-palm plantations established on land that lacked forest cover for at least five years (solid line) or at least ten years (dashed line) prior to planting, for Borneo (A), Indonesian Borneo (B) and Malaysian Borneo (C).**


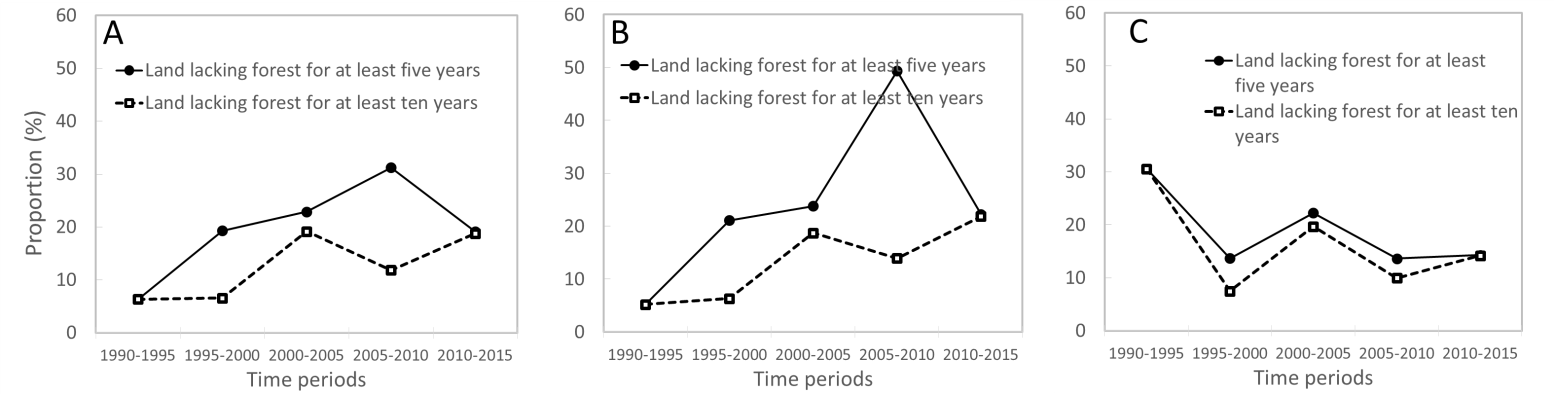


**Figure S5b│ Proportion (in area terms) of industrial pulpwood plantations established on land that lacked forest cover for at least five years (solid line) or at least ten years (dashed line) prior to planting, for Borneo (A), Indonesian Borneo (B) and Malaysian Borneo (C).**


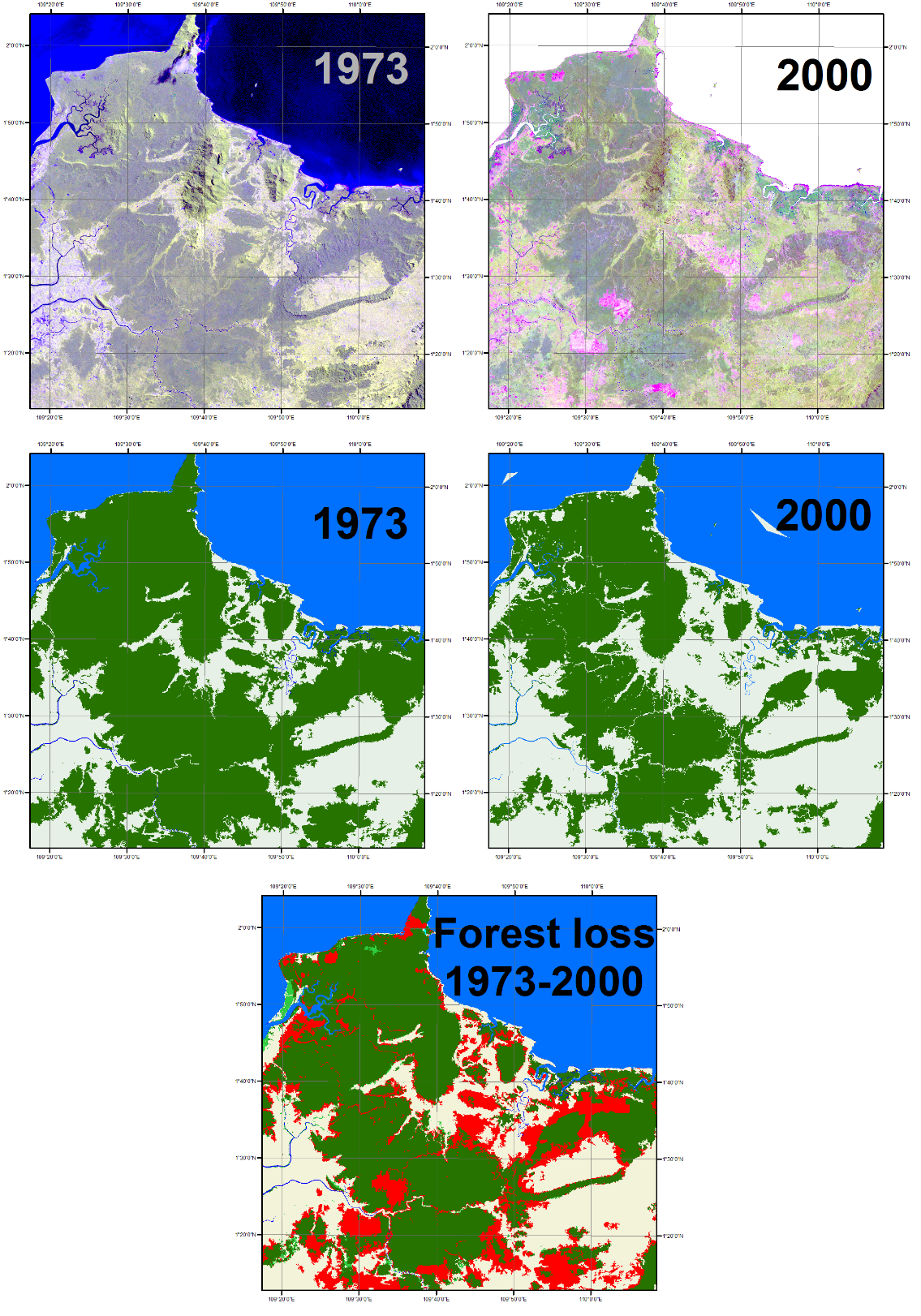


**Figure S6│ A close up view of an area in Western Borneo revealing losses in forest area from 1973 to 2000.** On the 1973 LANDSAT 1 imagery (false color composite: 3-4-2) used to create the 1973 map of old-growth forest (see Gaveau et al. 2014), a landscape of forest blocks (dark green) and non-forest areas (yellow to light green) are clearly visible. On the 2000 LANDSAT 5 imagery (false color composite: 5-4-3) used to create the 2000 map of old-growth and selectively logged forest (see Margono et al. 2014), the same landscape is visible, although some forest blocks have been lost (deforestation). Maps created using *ArcMap* v10.2.2 geospatial processing program <http://www.esri.com/software/arcgis/arcgis-for-desktop>.


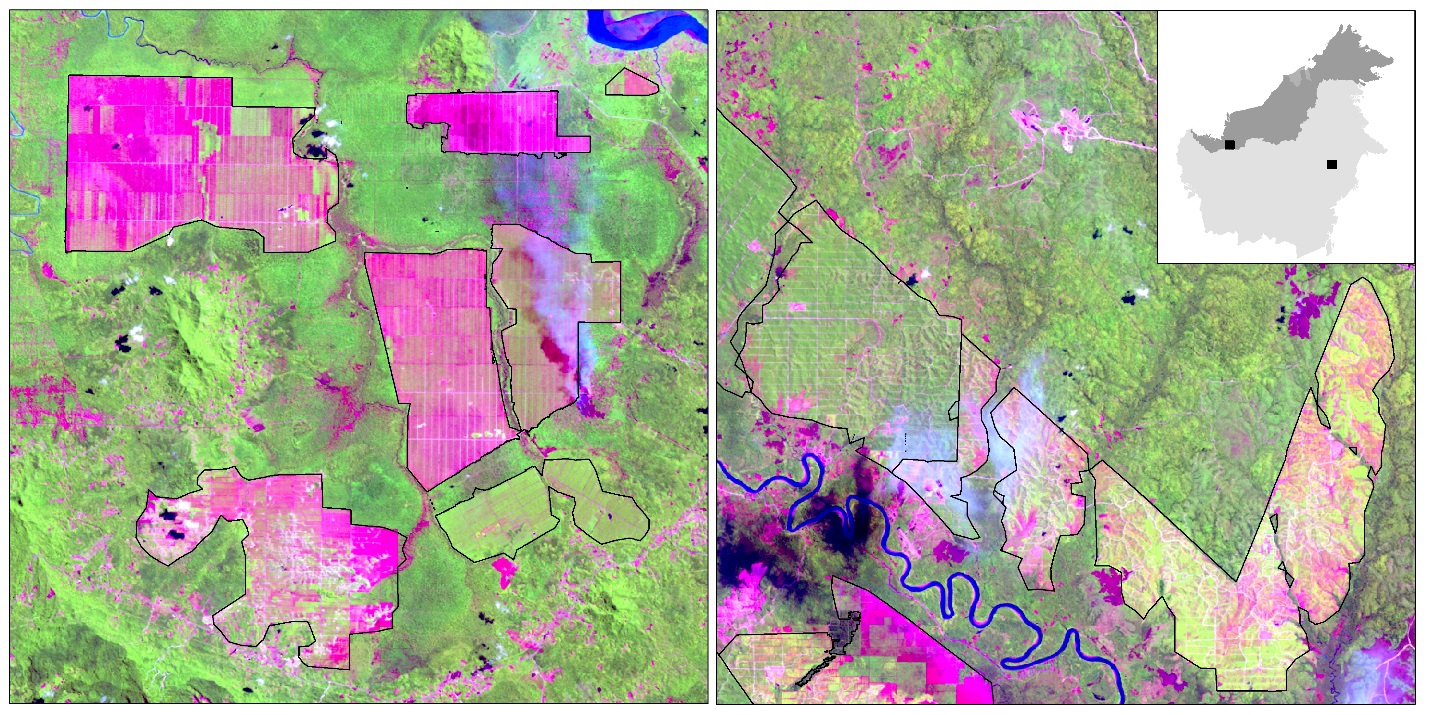


**Figure S7│ Close-up view (scale 1:100,000) of an area in Sarawak, Malaysian Borneo (Left Panel) and an area in East Kalimantan, Indonesian Borneo (Right Panel) (See top-right inset for locations).** On these LANDSAT 5 image snapshots acquired in year 2009 (false color composite: 5-4-3), industrial oil-palm plantations are easily recognized by their geometric shapes (rectangular-like shapes or otherwise more complex shapes with linear boundaries), their distinctive grid-like or contour-like patterns and by their homogeneous spectral colors characteristic of either young open-canopy (yellow), mature closed-canopy (green) single tree-species plantations, or recently-cleared lands devoid of vegetation (pink). Maps created using *ArcMap* v10.2.2 geospatial processing program <http://www.esri.com/software/arcgis/arcgis-for-desktop>.


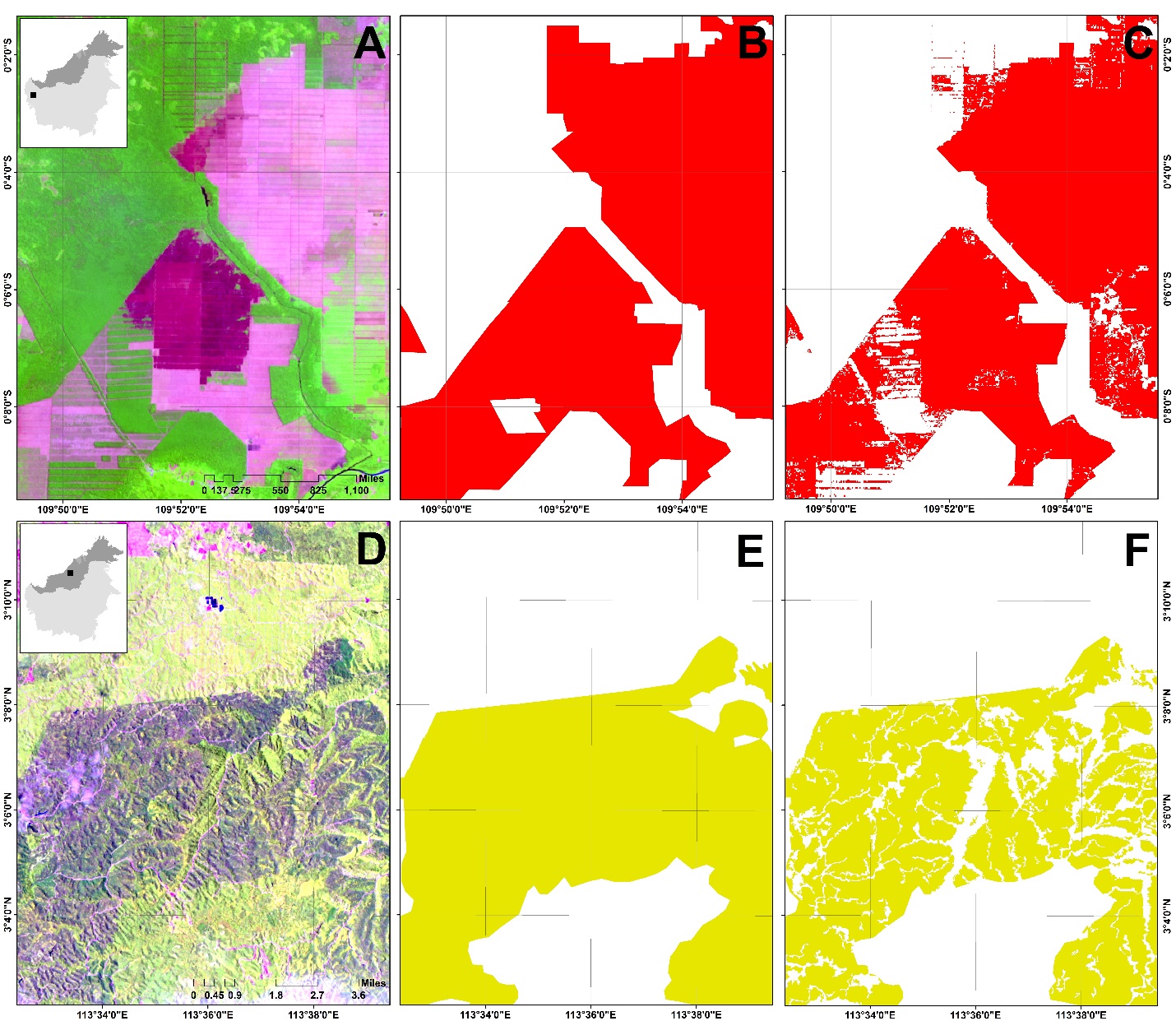


**Figure S8│ Examples of industrial oil-palm (A, B, C) and pulpwood (D, E, F) plantation extent before (B, E) and after (C,F) applying a correction procedure described in Methods.** Maps created using *ArcMap* v10.2.2 geospatial processing program <http://www.esri.com/software/arcgis/arcgis-for-desktop>.


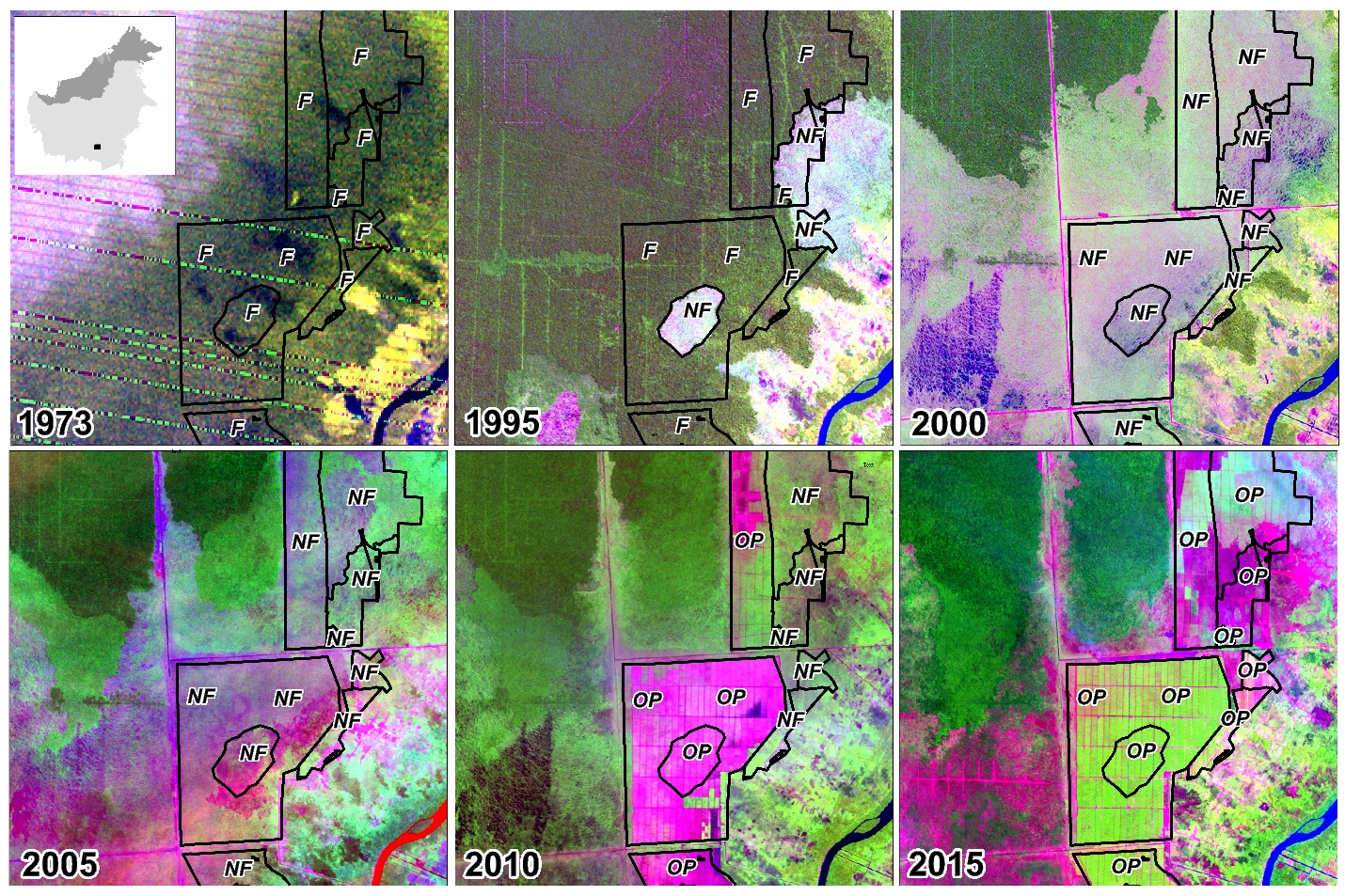


**Figure S9│ Close-up view (scale 1:65,000) of an area in Central Kalimantan Province, Indonesian Borneo (See top-left inset for locations) showing changes in forest cover since 1973 and before oil-palm plantations became established.** On these time-series LANDSAT snapshots (false color composite: 3-4-2 for 1973 MSS imagery; 5-4-3 for the 1995, 2000, 2005 and 2010 TM and ETM+ imagery; 6,5,4 for the 2015 OLI imagery), the forest area (*F*) appears dark green. In this example, much of the forest area has been converted to scrub lands (Non-forest; *NF*) by uncontrolled forest fires in 1997 before subsequently becoming converted to oil-palm plantations (*OP*). Note: in this example, much of the 1990 imagery was obscured by clouds, and hence several oil-palm polygons were first termed ‘uncertain’. Several polygons were subsequently recoded to ‘forest’ because the preceding and following years (1973 and 1995) indicated no change in forest cover. Maps created using *ArcMap* v10.2.2 geospatial processing program <http://www.esri.com/software/arcgis/arcgis-for-desktop>.


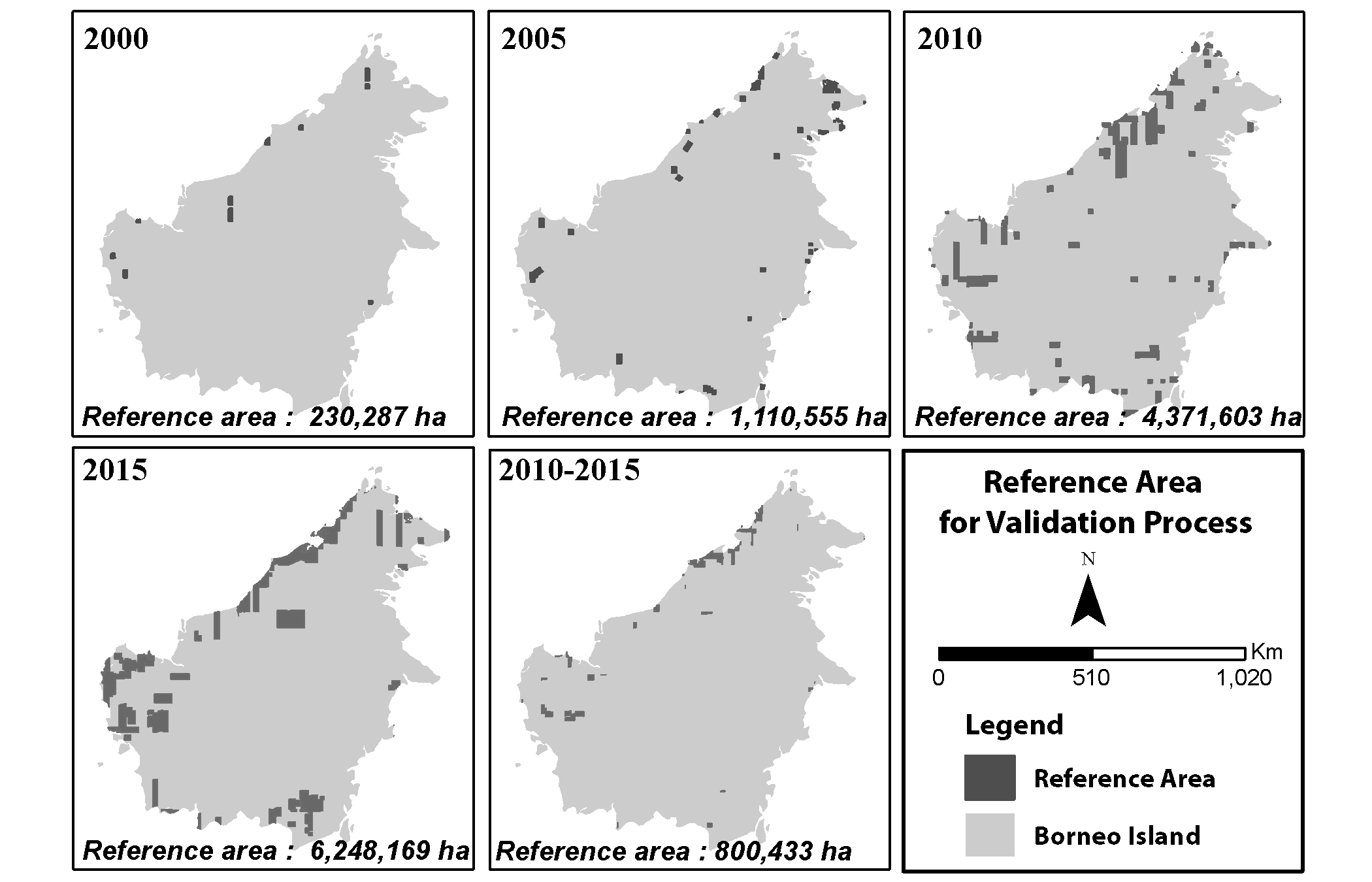


**Figure S10│ Map showing the reference areas where validation of the plantation maps was performed.** See Table S2-S6 for results of validation. Maps created using *ArcMap* v10.2.2 geospatial processing program <http://www.esri.com/software/arcgis/arcgis-for-desktop>.


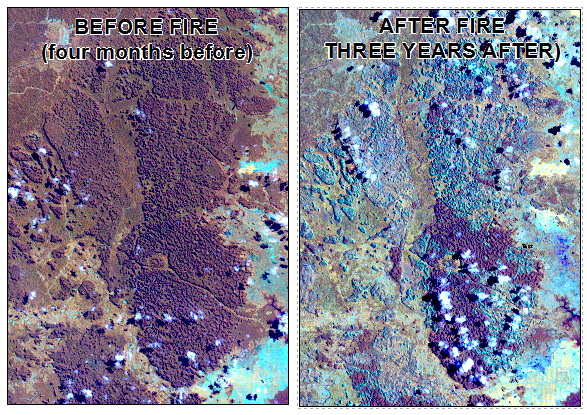


**Figure S11│ A close up view of an area in South Kalimantan province, Borneo where the 1997 ENSO-triggered drought and fires converted an intact forest to scrubs.** In the left panel, the forest was intact in May 1997, and appears dark brown on this LANDSAT 5 imagery (false color composite: 4-5-3). This forest burned in September-October 1997. In the right panel, three years after fire, much of this forest appears cyan, indicating areas with low vegetation cover. Maps created using *ArcMap* v10.2.2 geospatial processing program <http://www.esri.com/software/arcgis/arcgis-for-desktop>.

**References**

1 Carlson, K. M. *et al.* Carbon emissions from forest conversion by Kalimantan oil palm plantations. *Nature Climate Change* **3**, 283-287 (2013).

2 Gunarso, P., Hartoyo, M., Agus, F. & Killeen, T. Oil palm and land use change in Indonesia, Malaysia and Papua New Guinea (2013). At < <http://www.tropenbos.org/file.php/1343/4_oil_palm_and_land_use_change_gunarso_et_al.pdf>> Date of access: 02/02/2014.
